# *Drosophila* primary microRNA-8 encodes a microRNA-encoded peptide acting in parallel of *miR-8*

**DOI:** 10.1186/s13059-021-02345-8

**Published:** 2021-04-23

**Authors:** Audrey Montigny, Patrizia Tavormina, Carine Duboe, Hélène San Clémente, Marielle Aguilar, Philippe Valenti, Dominique Lauressergues, Jean-Philippe Combier, Serge Plaza

**Affiliations:** 1 Laboratoire de Recherche en Sciences Végétales, Université de Toulouse 3, CNRS UMR5546, 31320 Auzeville-Tolosane, France; 2 Laboratoire MCD, Centre de Biologie Intégrative, Université de Toulouse 3, CNRS UMR5077, Bat 4R4, 118 route de Narbonne, 31062 Toulouse, France

**Keywords:** *Drosophila*, sORF, lncRNA, *miR-8*, miPEP, Small peptides

## Abstract

**Background:**

Recent genome-wide studies of many species reveal the existence of a myriad of RNAs differing in size, coding potential and function. Among these are the long non-coding RNAs, some of them producing functional small peptides via the translation of short ORFs. It now appears that any kind of RNA presumably has a potential to encode small peptides. Accordingly, our team recently discovered that plant primary transcripts of microRNAs (*pri-miRs*) produce small regulatory peptides (miPEPs) involved in auto-regulatory feedback loops enhancing their cognate microRNA expression which in turn controls plant development. Here we investigate whether this regulatory feedback loop is present in *Drosophila melanogaster*.

**Results:**

We perform a survey of ribosome profiling data and reveal that many pri-miRNAs exhibit ribosome translation marks. Focusing on miR-8, we show that *pri-miR-8* can produce a miPEP-8. Functional assays performed in Drosophila reveal that miPEP-8 affects development when overexpressed or knocked down. Combining genetic and molecular approaches as well as genome-wide transcriptomic analyses, we show that *miR-8* expression is independent of miPEP-8 activity and that miPEP-8 acts in parallel to *miR-8* to regulate the expression of hundreds of genes.

**Conclusion:**

Taken together, these results reveal that several *Drosophila pri-miRs* exhibit translation potential. Contrasting with the mechanism described in plants, these data shed light on the function of yet undescribed *primary-microRNA*-encoded peptides in *Drosophila* and their regulatory potential on genome expression.

**Supplementary Information:**

The online version contains supplementary material available at 10.1186/s13059-021-02345-8.

## Background

More than 20 years after the first genome annotation, it is now becoming clear that the protein-centric view of gene content strongly underestimates the number of DNA regions that are expressed and fulfill important functions for development and physiology since the majority of the genome is in fact transcribed [1]. A first discovery was the importance of hundreds of small non-coding RNAs, such as microRNAs (*miR*), playing regulatory roles in the silencing of genes and transposable elements. More recently, genome-wide transcript profiling has disclosed the existence of numerous RNAs referred to as long non-coding RNAs (lncRNAs or lincRNAs) since they lack the classical hallmarks of protein-coding genes.

Although the functions of all lncRNAs remain largely unknown, there are several experimental cases illustrating their key role as functional RNAs in various steps of the control of genome expression [2]. In association with other molecules, lncRNAs can coordinate several physiological processes and their dysfunction impacts several pathologies, including cancer and infectious diseases. lncRNAs control genetic information, such as chromosome structure modulation, transcription, splicing, messenger RNA (mRNA) stability, mRNA availability and post-translational modifications. They also act as scaffolds, bearing interaction domains for DNA, mRNAs, miRs and proteins, depending on both their primary sequence and secondary structure [3]. In addition, while lncRNAs annotated as non-coding cannot produce large-sized proteins, they all contain myriads of short open reading frames (sORF) [4–7] and a surprising result was the discovery for a subset of them of their translation into small functional peptides [8–11].

MicroRNAs (miRs) define a class of small, non-coding RNAs able to downregulate the expression of target mRNA by binding to the 3′-ends inducing mRNA degradation and/or translation repression. Intergenic microRNAs are produced from the sequential cleavage of long precursors named primary transcripts of microRNA (*pri-miRs*) (frequently annotated as lncRNAs) by Drosha and Dicer into 22 nt miR duplexes associated with the RISC protein complex. Identified in a broad spectrum of living species, they are transcribed from coding genes or lncRNAs by the RNA polymerase II. MiRs are critical for normal animal development and are involved in many biological processes [12]. Due to their role in silencing, *miRs*, and in particular the *pri-miR*s they come from, have always been considered as non-coding. However, the discovery that plant *pri-miR*s encode small regulatory peptides (miPEPs) merely adds evidences that *pri-miRs* are also pre-mRNAs [13]. In plants, miPEPs specifically increase transcription of their primary transcript impacting the level of the mature miR produced and consequently affecting the control of the entire *miR* Gene Regulatory Network (GRN). To date, this regulation has been extended to several miRs in various plants [14–17]. In human cells, only few reports present evidences of pri-miR translation [18–21]. Pri-miR-22 host gene endogenously produces a miPEP for which the function is unknown [19]. miR-200 might produce a miPEP able to control the Vimentin, a miR200 target [18]. miPEP155 was described to control major histocompatibility complex class II-mediated antigen presentation by disrupting the HSC70-HSP90 machinery [20]. However, whether these miPEPs control the expression of their cognate miR was not investigated. More recently, miPEP133 was discovered in miR34a as a tumor suppressor localized in the mitochondria. It enhanced p53 transcriptional activation which, in turn, induces miR-34a expression [21]. In summary, whereas few human pri-miRs appear translatable, it remains not clear whether the regulation found in plants exists in animals. Here we addressed this question in *Drosophila melanogaster*. We show that several intergenic *pri-miRs* contain marks of ribosome profiling. To investigate whether *Drosophila* can produce miPEPs, we focused on *miR-8*, a previously well-characterized microRNA that sustains many developmental traits [22]. *miR-8* controls organism physiology, tissue growth and survival [23–26], stem cell renewal [27–30], central nervous system development [30–32], signaling and developmental pathways [33–41]. Consequently, *miR-8* loss or gain of function impinges on fly development and survival. In the *pri-miR-8*, we located a small ORF encoding a potential 71 amino acid peptide we called miPEP-8. We showed that this sORF is translatable in vitro and in vivo. While our attempts to reveal an auto-regulatory loop remained unsuccessful, we showed by genetic and transcriptomic approaches that miPEP-8 and *miR-8* act in parallel in controlling wing development and in regulating the expression of distinct sets of genes.

## Results

### Translation potential of sORFs present in *Drosophila pri-microRNAs*

**Fig. 1:**
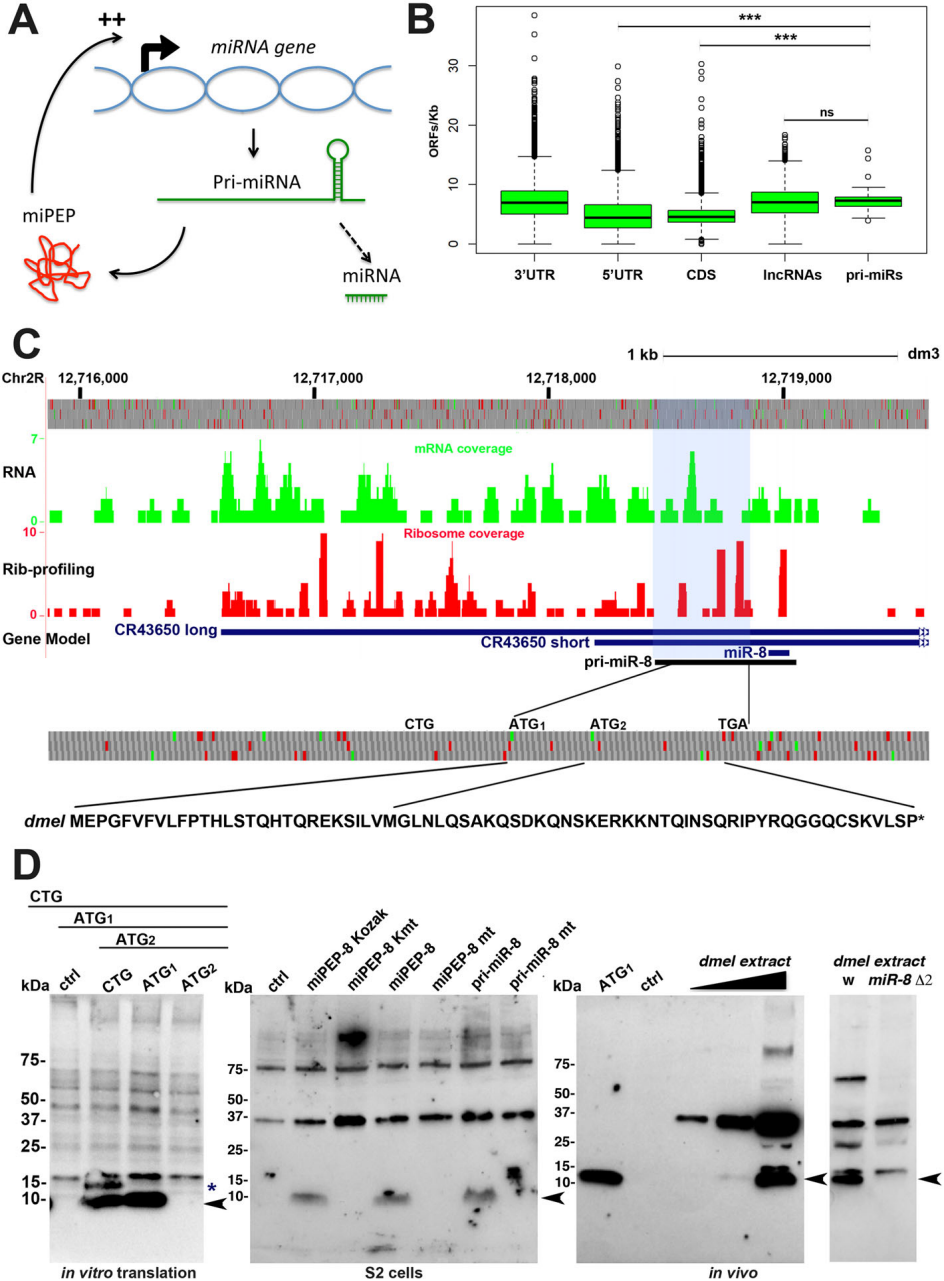
Translatability of *pri-miR-8*. **a** Model of miPEP regulation in plants. **b** Box plot representation of the number of ORFs in different classes of RNAs. 3′UTR, 5′UTR and CDS represent coding RNAs, whereas lncRNAs and pri-miRs represent non-coding RNAs. An ORF was defined as starting with an ATG and coding for a minimum of 10 amino acids. *Pri-miRs* reveal comparable numbers of ORFs/kb as lncRNAs. **c** GWIPS-vis [42] genome viewer of the *Drosophila miR-8* locus. Top: genomic positions and ORFs in the three reading frames. Green bars define ATGs and red bars stop codons. Below, RNA-seq profile is shown in green and ribosome profiling is shown in red. The blue horizontal lines represent the two CR43650 non-coding RNA transcripts and potential *miR-8 pri-miRs*. In black is schematized the transcript we identified as detectable *pri-miR-8*. Bottom: miPEP-8 amino acid sequence is shown. **d** Western blot experiments using the anti-miPEP-8 antibody. Left panel: in vitro synthetized miPEP-8 corresponding to the constructs indicated on top. The asterisk indicates the upstream initiated peptide. The arrow indicates the miPEP-8 initiated at ATG1. Middle panel: detection of miPEP-8 in S2 cells over-expressing miPEP-8 placed in different translational contexts; Kozak (optimal); K mt (ATG mutated into TGA); mt (ATG mutated into AGT). Note that the *pri-miR* is translated and endogenous miPEP-8 expression is undetectable in S2 cells. Right panel: anti miPEP-8 western blot of adult *Drosophila* extracts and in the *miR-8* deleted line Δ2/Δ2 [26] in which no miPEP-8 is detected. We noticed the presence of non-specific bands as well as additional specific bands representing possibly miPEP-8 multimeric forms or PTM modifications. Ctrl corresponds to cell extracts transfected with an empty vector

As plants *pri-miRs* contain sORFs (miORFs) producing functional miPEPs involved in a positive feedback loop on *pri-miR* expression (Fig. 1a), we first asked whether *D. melanogaster pri-miRs* contain significant levels of miORFs. We scored the number of sORFs present in intergenic *miR* genes and their *pri-miR* and compared this number with other classes of RNAs, coding as well as non-coding (Fig. 1b). *Pri-miRs* present the highest enrichment of sORFs/kb when compared with the 5′UTR of coding genes, sequences known to contain translatable short open reading frames, but contain similar amounts of sORFs when compared with lncRNAs, previously reported to be widely bound by ribosomes [43]. Ribosome profiling experiments were developed to study translatability of RNAs by scoring the sequences bound by ribosomes [7, 44, 45] including studies conducted on *Drosophila* [4, 46, 47]. We searched if and how many *Drosophila pri-miRs* were widely bound by ribosomes. Briefly, we searched in the rib-seq databases marks of ribosome binding in predicted *pri-miR*. To avoid difficulties for the interpretation with *miRs* embedded in host coding genes, we focused only on the intergenic *miRs*. We found many marks of ribosome profiling and identified hundreds of potentially translated sORF peptides within dozens of *pri-miRs*, suggesting that, as observed in plants, *Drosophila pri-miRs* are potentially translated (Additional file 1: Fig. S1; Additional file 2).

### *Drosophila pri-miR8* encodes a miPEP-8 translated in vivo

To further characterize the potential translation of *pri-miRs*, we focused on the *miR-8* primary transcript (*pri-miR-8*). *MiR-8* is an intergenic miR and is likely produced from the expression of long non-coding CR43650 spanning over the *pre-miR-8* (Fig. 1c). In flybase, two CR43650 ncRNAs were predicted, a long and a short form defining putative *pri-miR-8* transcripts, independently identified by two different teams and likely initiated from different promoters [48, 49]. As shown in Fig. 1c, many marks of potential translation were found along the CR43650 transcripts. We first performed 5′RACE as well as RT-PCR assays to determine which isoform was preferentially produced. While we successfully detected the short isoform in flies and S2 cells, we did not succeed in amplifying the long isoform, suggesting that the short transcript is the most abundant isoform expressed, a result confirmed by RNA-seq data generated during this study (Additional file 1: Fig. S2 and S3A). Our RNA-seq data further suggest that the long transcript might define a different transcription unit since no overlapping reads were detected in the promoter region of the short transcript (Additional file 1: Fig. S3A). In agreement with this, a GAL4 enhancer trap recapitulating *miR-8* expression is inserted just upstream of the short transcript [24]. These flies express *miR-8* at a level comparable to control flies (Additional file 1: Fig. S3B), showing that this insertion disrupting the co-linearity of the *miR-8* locus is not detrimental for *miR-8* expression. Finally, we verified that this short transcript (referred hereafter as *pri-miR-8*) is functional since it efficiently produces a functional mature *miR-8* able to down regulate a *miR-8* sensor (see below Fig. 3b, d).

We therefore looked for a potential open reading frame within the *pri-miR-8* gene. Focusing on the 5′ leader sequence of *pri-miR-8*, we found one ORF located upstream of the *pre-miR-8.* This ORF is the longest ORF present 5′ to the *pre-miR*, which potentially encodes a miPEP of 71 amino acids in length if initiated from the first ATG (ATG1) (Fig. 1c and Additional file 1: Fig. S2A). However, the presence of a second ATG (ATG2), located downstream, gives the possibility to produce a shorter peptide. To determine whether the open reading frame is translated and which initiation codon is used, we generated and characterized specific antibodies (Additional file 1: Fig. S4). In parallel, we generated different deletion constructs and performed in vitro translation experiments using insect cell extracts. As shown in Fig. 1d (left panel), we observed an efficient translation from the longest construct (CTG) consisting in an extended genomic region of the defined 5′ leader sequence of *pri-miR-8.* Deletion experiments revealed a stronger and efficient translation from ATG1 but not from ATG2. A higher product, possibly initiated at an upstream codon present in the construct was also detected but was not further investigated since it was not present in *pri-miR-8*.

We next generated translatable and untranslatable miPEP-8 forms placed in optimal translational Kozak (K) or mutated kozak (KMT) contexts or in the miPEP-8 natural ATG1-initiated translational context. We then expressed these miPEP-8 constructs in *Drosophila* S2 cells (Fig. 1d, middle panel). As revealed by western blot experiments, these different constructs produced the same level of miPEP-8 when the ATG was placed in an optimal or in its natural translational context whereas no miPEP-8 was detected from mutated ATG constructs. This result reveals that the natural nucleotide context of miPEP-8 miORF is in a favorable translational context. We next questioned whether *pri-miR-8* was able to produce miPEP-8. We transfected S2 cells with wild type or ATG-mutated *pri-miR-8* constructs and performed western blot experiments. We observed that only the wild-type *pri-miR-8* was able to produce miPEP-8. Finally, we examined whether a peptide corresponding to miPEP-8 was detectable in fly extracts by performing a western blot experiment on young adult flies. As revealed in Fig. 1d (right panel), we observed a signal co-migrating with the in vitro synthetized miPEP-8, corresponding to endogenous miPEP-8 as demonstrated by the lack of this band in *miR-8* deleted Δ2 mutant flies. Sequence alignment analyses revealed that this peptide is poorly conserved among *Drosophila* species. Some homologies are detected in *Drosophila melanogaster* group but no conservation was found in more distant *Drosophilae* species.

Altogether, our results reveal that the *pri-miR-8* transcript carries, in addition to the *miR-8* sequence, at least one translated ORF located upstream of the *pre-miR*, able to express a miPEP-8 both in vitro and in vivo.

### Expression of miPEP-8 impinges on *Drosophila* development

**Fig. 2:**
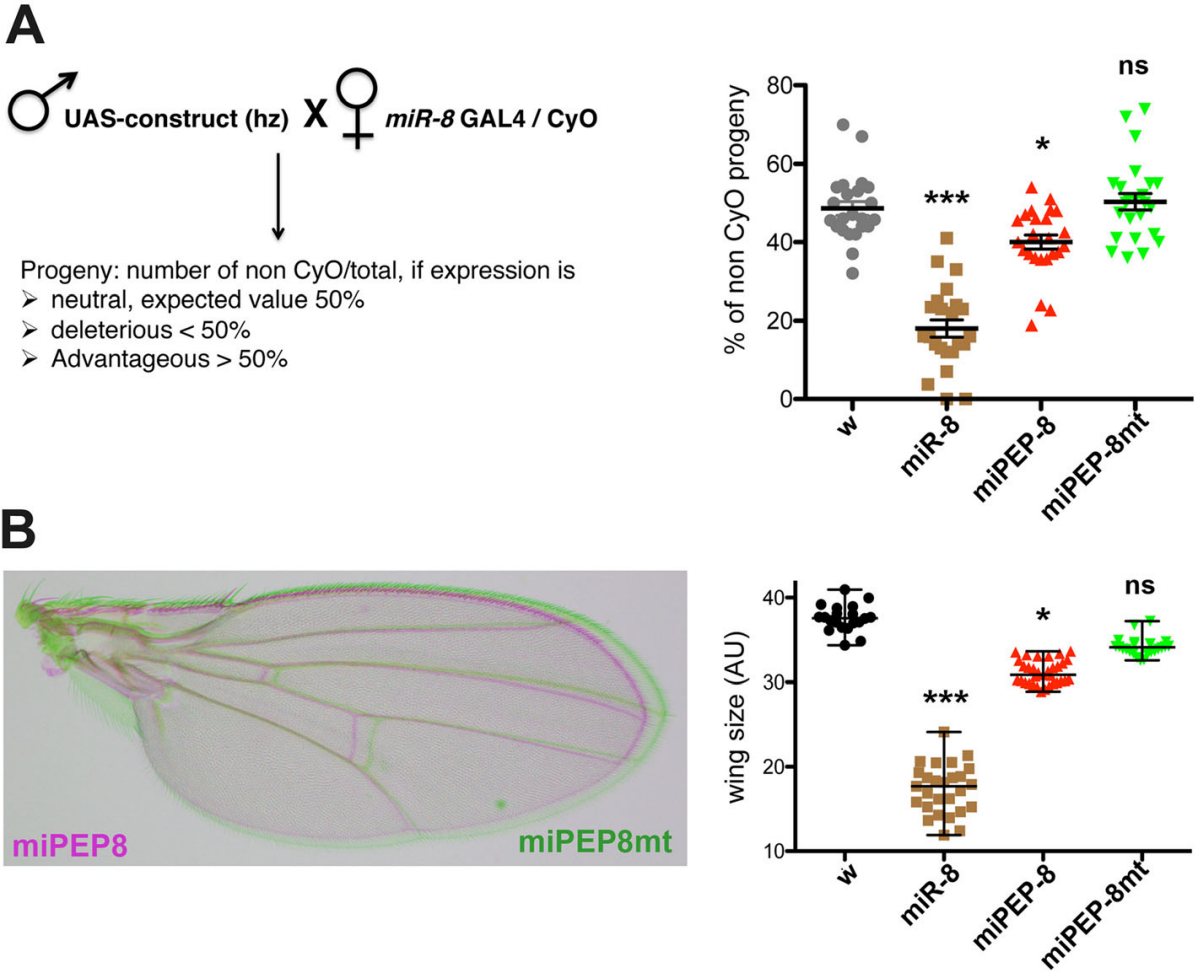
*Drosophila* miPEP-8 is biologically active during development. **a** Lethality assay on flies over-expressing *miR-8*, miPEP-8 or miPEP-8mt (ATG mutated) using the *miR-8* GAL4 driver. Left: details of the genetic cross and expected percentage depending on the effect (neutral, deleterious or advantageous) on *Drosophila* development. Right: graph indicating the percentage of hatched flies over-expressing the different constructs. *White* flies (*w*) crossed with the driver line were used as a control. Expressing *miR-8* resulted in developmental lethality since less than 20% of flies hatched (expected value 50%). A significant decrease occurred following miPEP-8 over-expression but not with the untranslatable miPEP-8mt construct. Number of independent crosses: for *w* and *miR-8 n* = 23; for miPEP-8 wt and mt *n* = 24. **b** Same as in **a** except the constructs were expressed in wings using the MS1096 driver and the phenotypes scored on wing size. Number of wings analyzed: for *w n* = 20; for *miR-8 n* = 27; for miPEP-8 wt and mt *n* = 27. * or ns: Significant differences are indicated relative to white recipient flies. AU arbitrary units

To study the function of miPEP-8, we generated flies able to express a translatable and untranslatable version of miPEP-8 (ATG mutated). *miR-8* sustains many biological functions in *Drosophila* and either its loss of function or its over-expression leads to detrimental outcomes in cells, tissues or the whole organism [23–27, 29, 31–37, 40, 41, 50], providing a useful readout to assess miPEP-8 activity. To test our hypothesis that miPEP-8 controls *miR-8* expression and modulates its activity, we used an over-expression assay. Using the *miR-8* GAL4 driver, a GAL4 insertion in the endogenous *miR-8* promoter reported to mimic *miR-8* expression [23, 24, 33, 40], we first asked whether over-expression of miPEP-8 impinges on fly viability. As reported, driving UAS-*miR-8* over-expression results in increased fly lethality [23] (Fig. 2a). Over-expression of a UAS-miPEP-8 translatable construct also affected fly viability whereas the untranslatable form did not. This indicates that, like *miR-8*, the translatable form of miPEP-8 is able to interfere with development, although with a weaker effect compared to *miR-8*.

By loss or gain of function experiments, *miR-8* was shown to induce a « small wing » phenotype [24, 26, 40]. We therefore questioned whether over-expression of miPEP-8 also induced a wing phenotype (Fig. 2b). Using the wing driver line MS1096, *miR-8* over-expression induced several wing defects, from a reduced size to the loss of wing vein, sensory organs, miss shaped, depending on the transgene/promoter strength [40, 41]. Quantifying the wing size appeared the most reliable criteria, and we compared this phenotype with the phenotype observed with miPEP-8 over-expression. Consistently with our above result, miPEP-8 over-expression induced a slight, albeit significant wing size reduction, revealing yet again a weaker activity compared with *miR-8* (Fig. 2b). Importantly, miPEP-8-induced wing reduction was dependent on the integrity of the translation codon since the same construct with the mutated ATG did not induce any phenotype (Fig. 2b).

Altogether, these experiments show that miPEP-8 appears to be biologically active but induces a milder phenotype compared to *miR-8*.

### In *Drosophila*, *miR-8* expression is independent of miPEP-8 expression

**Fig. 3:**
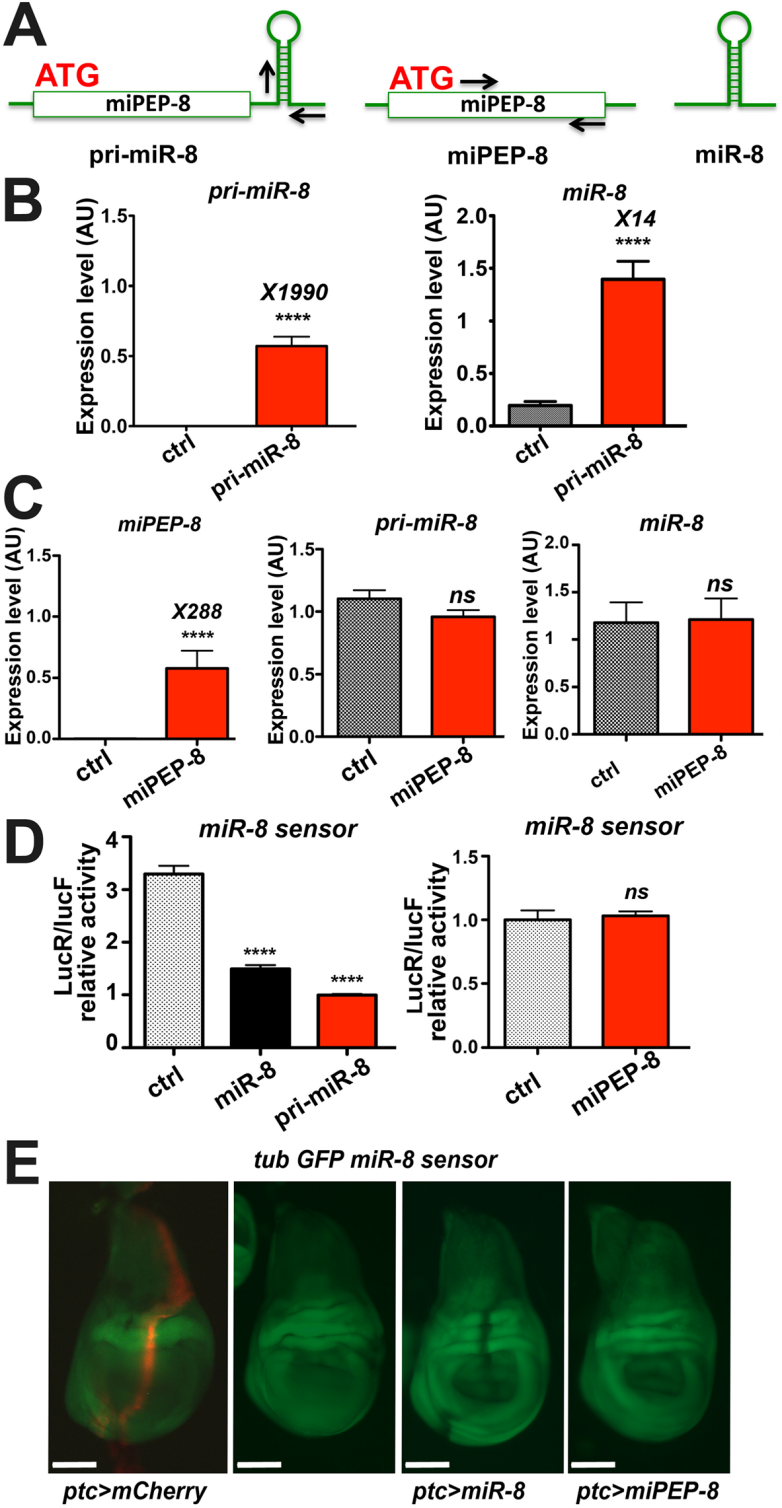
*Pri-miR-8* expression is independent of miPEP-8 control/activity. **a** schematic representation of constructs tested on *miR-8* expression and activity levels. Arrows locate the primers used in the qPCR experiments determining miPEP and *pri-miR* relative expression levels. **b** the characterized *pri-miR-8* produces a mature *miR-8*. S2 cells were transfected with a vector expressing *pri-miR-8*. Left: detection of the over-expression level of *pri-miR-8* by qPCR. Right: detection of the over-expression level of mature *miR-8* using the same RNA samples, *n* = 11 **c** miPEP-8 lacks repressive activity towards *miR-8* expression. Left: level of miPEP-8 over-expression. Middle and right panels: quantification of *pri-miR-8* and mature *miR-8* in miPEP-8 over-expressing cells compared to control transfected cells (ctrl). *n* = 13 for the ctrl and 14 for miPEP-8. **d**, **e** Insensitivity of *miR-8* sensor to miPEP-8 over-expression in S2 transfected cells (*n* = 16) (**d**) or in wing imaginal discs when miPEP-8 is expressed under the *ptc*-GAL4 promoter (**e**). In **d**, a *miR-8* construct (*n* = 12) [17] was used as a positive control repressing the *miR-8* luciferase sensor [20]. Of note, *pri-miR-8* (*n* = 21) also repressed the *miR-8* luciferase sensor. In **e**, first panel to the left: *ptc* GAL4 crossed with a UAS mCherry. Second panel to the left: expression pattern of the GFP *miR-8* sensor alone. Scale bars (white) indicate 100 μm. A repressive activity is observed with *miR-8* expressed in the *ptc* domain but not with miPEP-8. A representative disc is shown out of ten analyzed

In plants, miPEPs positively auto-regulate the expression of their own *miR* by regulating the expression of their cognate *pri-miR*. To test whether miPEP-8 regulates *miR-8* expression in *Drosophila*, we monitored the level of *pri-miR-8* and *miR-8* through quantitative PCR experiments on S2 cells and on flies expressing the translatable form of miPEP-8. We first set up experimental conditions in S2 cells by transfecting the *pri-miR-8* construct and quantified the level of the exogenous *pri-miR* and mature miR produced. When *pri-miR-8* was over-expressed, both over-expression of *pri-miR-8* and *miR-8* was detectable (Fig. 3b). We then over-expressed miPEP-8 and quantified the endogenous level of *pri-miR-8* and mature *miR-8*. Whereas we unambiguously detected the over-expression of the miPEP-8 construct, we did not see any change in the levels of endogenous *pri-miR-8* or mature *miR-8* expression (Fig. 3c). As observed in S2 cells, no change in *pri-miR-8* or miR-8 levels was observed in flies upon miPEP-8 expression using the *miR-8* GAL4 driver (Additional file 1: Fig. S5).

We next questioned the potential regulatory role of miPEP-8 on *miR-8* expression by testing miPEP-8 overexpression on endogenous miR-8 activity level in the presence or absence of. One way of challenging this question is the use of a sensor of miR-8 activity, whether endogenous or resulting from over-expression. Thus, we designed a miR-8 luciferase reporter, bearing a 3′UTR from the *escargot* gene (*esg*), previously shown directly regulated by *miR-8* [27]. Over-expression of *pri-miR-8* in S2 cells was able to repress the miR-8 sensor to the same extent as *miR-8* (Fig. 3d, left panel), hence validating our miR-8 sensor. As mentioned above, this also indicates that the *pri-miR-8* construct is able to generate a functional and mature *miR-8*. Clearly, however, over-expression of miPEP-8 did not reveal any modulation of the luciferase reporter (Fig. 3d right panel). We performed similar experiments in vivo using a miR-8 GFP sensor in wing imaginal discs where *miR-8* was previously shown to be functional [33]. Expressing *miR-8* under the patched (ptc) GAL4 promoter led to the repression of the GFP in the ptc domain (Fig. 3e ptc > miR8 panel). Consistently, miPEP-8 over-expression had no effect on the miR-8 GFP sensor in vivo (Fig. 3e ptc > miPEP-8 panel). Altogether, these results indicate that miPEP-8 is not able to control *miR-8* expression or activity for that matter (see more below). We obtained similar conclusions on endogenous miR-8 target in S2 cells and in wing discs (Additional file 1: Fig. S6).

Finally, we asked whether the miPEP-regulation of *pri-miR* observed in plants is system specific by testing whether a plant *pri-miR*, upregulated by its miPEP in plant cells, could be upregulated in *Drosophila* cells. Reciprocally, we tested whether miPEP-8 is able to upregulate its *pri-miR-8* in plant cells. To that end, we expressed the plant *Arabidopsis thaliana pri-miR165a* and miPEP-165a in S2 cells using the actin promoter and measured the level of *pri-miR* produced in the absence or presence of the miPEP. Reciprocally, the *Drosophila pri-miR-8* and its miPEP-8 were cloned in plant expression vectors and agroinfiltrated in *Nicotiana benthamiana* leaves as performed previously [13]. Whereas the upregulation of the *A. thaliana pri-miR-165a* by miPEP-165a was observed in *N. benthamiana*, we did not detect any upregulation, but rather a downregulation of *pri-miR-165a* in *Drosophila* S2 cells (Additional file 1: Fig. S7). Reciprocally, we could detect a slight but significant increase of *pri-miR-8* expression upon miPEP-8 over-expression in *N. bentamiana* leaves, suggesting that a difference of regulation occurs between plant cells and insect cells (Additional file 1: Fig. S7). Although, *miR-8* expression appears to be miPEP-8 independent in *Drosophila*, these results further suggest that, like for plants miPEPs, animal miPEPs might nonetheless have the potential of autoregulating the expression of their cognate pri-miR.

### Endogenous miPEP-8 alteration reveals in vivo activity

**Fig. 4:**
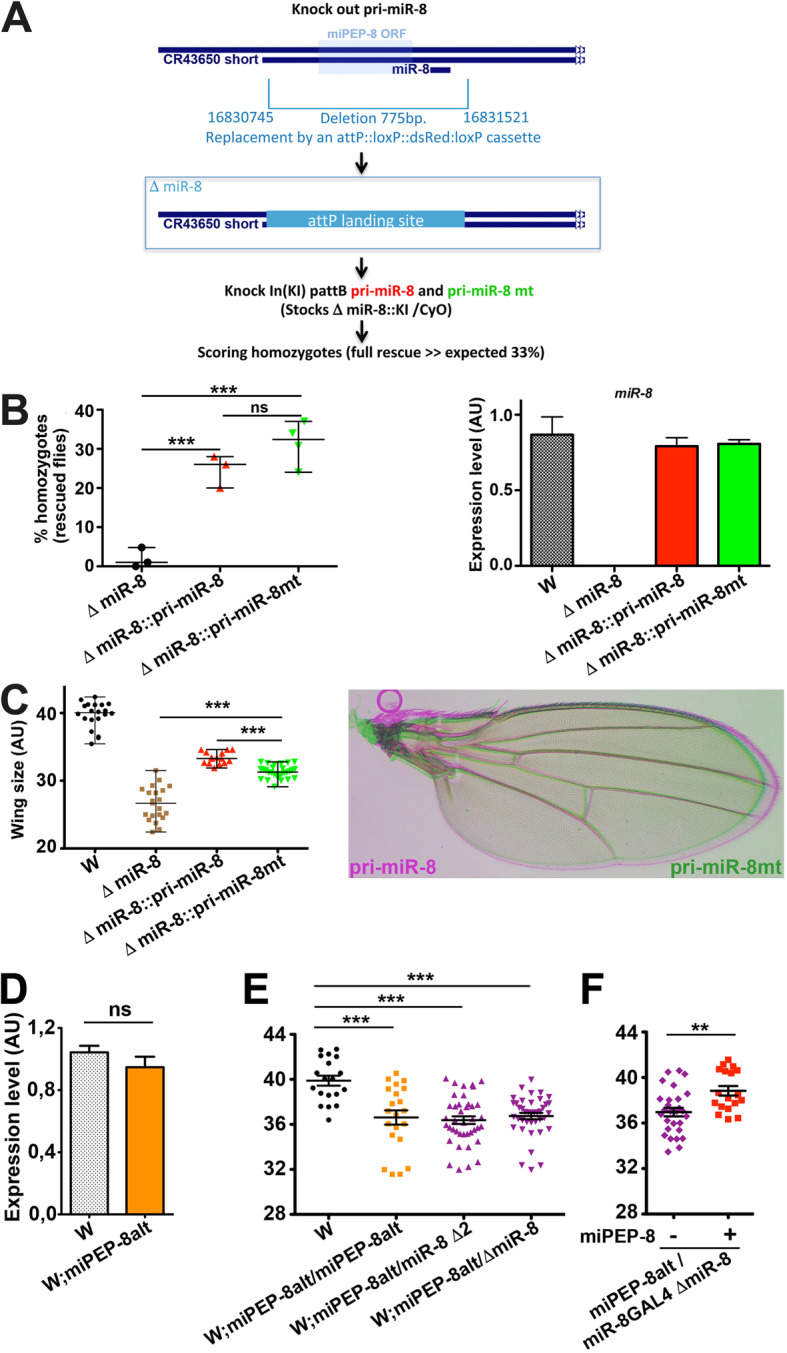
targeting miPEP-8 in vivo in *Drosophila* induces a wing phenotype*.* **a** Strategy for endogenous miPEP-8 edition. The *pri-miR-8* gene region was deleted by CRISPR and a P landing site was created. Wild type and miPEP-8 ATG mutated *pri-miR-8* in pattB were inserted at the P landing site. **b** Similar rescue efficiency was observed in at least three independent transgenic lines (left panel). qPCR on mature miR-8 in wild type and mutant (mt) *pri-miR-8* Knock In (KI) lines showed similar miR-8 levels (*n* = 4), (right panel). **c** Wing phenotype in *miR-8* deletion edited line. The *pri-miR-8* miPEP-8 mutated (mt) shows a reduced wing size compared to the wild type *pri-miR-8*. (*n* = 15 and 28 respectively). d–f Analyses in miPEP-8 mutant identified in DGRP polymorphism. **d** miR-8 level determined by qPCR in white recipient flies (w) and in white flies carrying the miPEP-8 truncated form (miPEP-8alt), (*n* = 6 and 8, respectively). **e**, **f** Wing size determination in different genetic contexts. miPEP-8alt homozygotes or over *miR-8* deficiencies revealed significant reduced wing size relative to the white recipient flies (w, *n* = 19; miPEP-8alt, *n* = 21; miPEP-8alt/miR-8 deletions, *n* = 40). Expressing miPEP-8 rescued the wing phenotype of miPEP-8alt flies relative to sibling flies not expressing miPEP-8 (*n* = 18 and 28, respectively). Significant (*) or nonsignificant (ns) differences are indicated either relative to white recipient flies or between the two groups

To investigate the functional requirement of miPEP-8 *in Drosophila*, we tried several times to edit the miPEP-8 in flies using CRISPR/Cas9, but unsuccessfully. In contrast, it was possible from the first attempt to delete the entire *miR-8* locus, showing that the failure to obtain a specific miPEP-8 edited line is not due to trivial technical problems. We therefore created a specific P landing platform in place of *pri-miR-8* transcript to perform Knock In strategies (Fig. 4a and Additional file 1: Fig. S8). This edited line exhibits the previously *miR-8* reported phenotypes [26], including a strong developmental lethality with only few escaping flies exhibiting a reduced size (including wings (Fig. 4c and Additional file 1: Fig. S8)) and leg defects (not shown). We further knocked in the wild type *pri-miR-8* and the *pri-miR-8* miPEP-8 untranslatable form (mt) at the P landing site and analyzed the outcomes. For both constructs, we observed a nearly total rescue since the theoretical expected 33.3% homozygotes (and 66.6% of CyO flies) in the progeny was almost reached (Fig. 4b left panel). These rescued flies appeared phenotypically normal and re-expressed *miR-8* at levels close to *miR-8* endogenous expression (Fig. 4b right panel). Both pri-miR-8 constructs restored significantly wing sizes when compared to the *ΔmiR-8* CRISPR line (Fig. 4c). Interestingly, a significant difference was observed in wings between the *pri-miR-8* wild type construct and the *pri-miR-8 mt* in which the miPEP-8 translatability was disrupted (Fig. 4c).

As a second approach, we took advantage of a polymorphism mutation detected in *Drosophila* Gene Reference Panel (DGRP) lines generating a premature stop codon leading to a 24 amino acid C-terminal miPEP-8 truncation called miPEP-8alt [51]. We outcrossed the miPEP-8alt DGRP line into white background and analyzed the consequence of the miPEP-8alt mutation. Whereas no significant difference was observed for miR-8 level between these the two miPEPs variants (Fig. 4d), flies homozygous for miPEP-8alt exhibit a significant wing size reduction when compared with the white flies expressing the miPEP-8 (Fig. 4e). We further analyzed the resulting wing phenotypes in different genetic contexts. The phenotype is also present when miPEP-8alt mutation was tested over a deletion of the *miR-8* gene (the Δ2 and the Δ*miR-8* CRISPR line generated in this study) (Fig. 4e), suggesting that the observed phenotype is a consequence of miPEP-8 loss of function. To test this, we performed rescue experiments by expressing miPEP-8 using the *miR-8* GAL4 driver line in miPEP-8alt/Δ*miR-8* background (Fig. 4f). Flies expressing miPEP-8 in miPEP-8alt/Δ*miR-8* CRISPR restored the wing size phenotype contrasting with the sibling control flies carrying no miPEP-8 transgene (absence of expression of wild type miPEP-8) (Fig. 4f).

Therefore, altogether, these experiments revealed an in vivo miPEP-8 function.

### The function of miPEP-8 is uncoupled from *miR-8* expression and activity

**Fig. 5:**
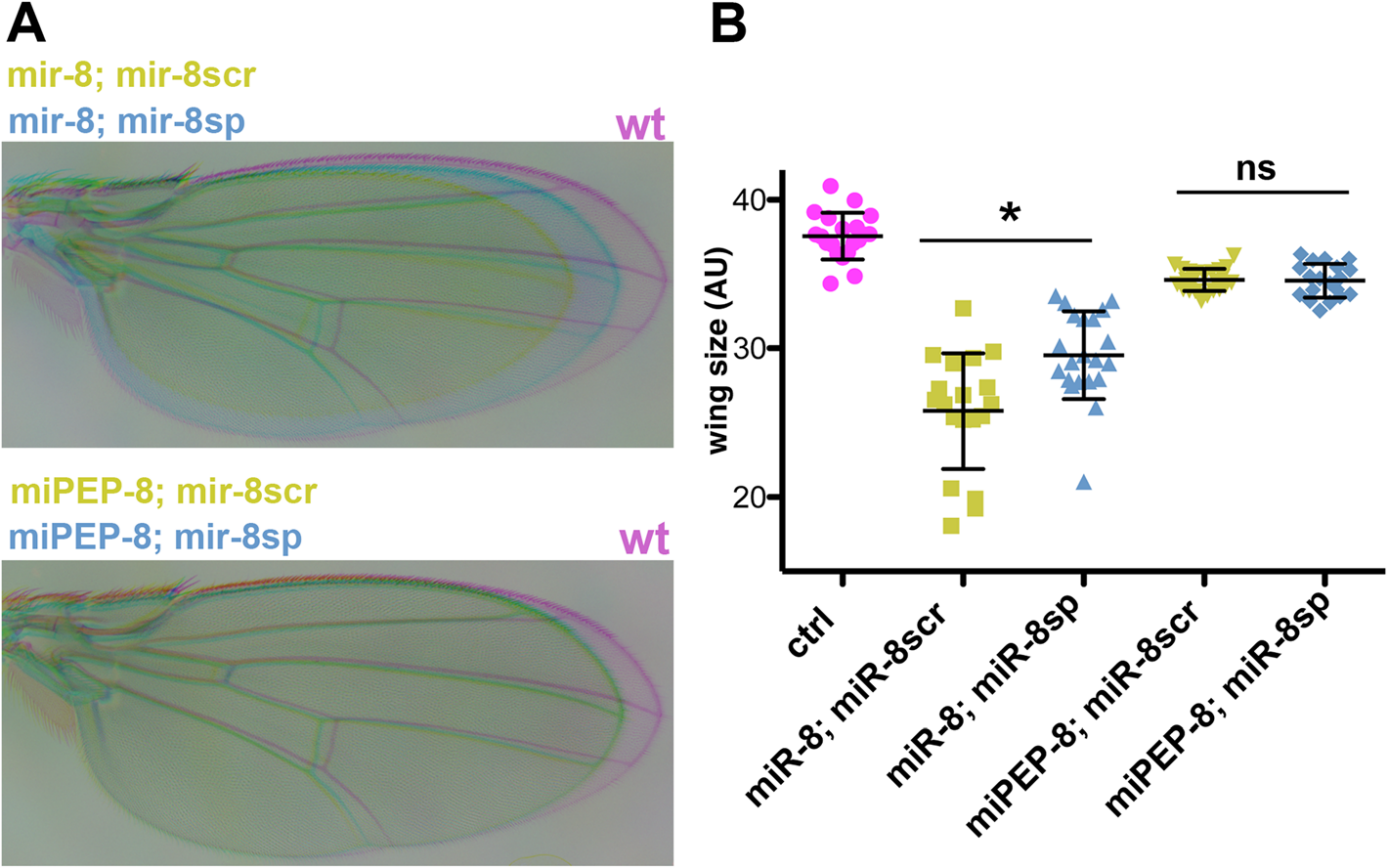
Uncoupled activity of miR-8 and miPEP-8. **a** Rescue assay of *miR-8*- or miPEP-8-induced wing phenotype in flies co-over-expressing *miR-8* or miPEP-8 along with a miR-8 sponge (miR-8sp) or a miR-8 scramble (miR-8scr). Only miR-8-sp (and not miR-8scr) compensates for *miR-8*-induced wing size reduction, hence efficiently titrating *miR-8*, while it has no effect on miPEP-8-induced wing phenotype. **b** Quantification of **a**. “ctrl” (MS1096/+) *n* = 19; “mir-8; mir-8scr” *n* = 20; “mir-8; mir-8sp” *n* = 21; “miPEP-8; mir-8scr” *n* = 23; “miPEP-8; mir-8sp” *n* = 19. * *p* < 0.05

The above experiments suggest that in *Drosophila*, miPEP-8 is not involved in a positive auto-regulatory feedback loop as observed in plants. However, due to the similarities of the phenotypes observed between *miR-8* and miPEP-8, we questioned whether miPEP-8 could be involved in the *miR-8* pathway through another mechanism or whether it acts in parallel of miR-8. As both *miR-8* and miPEP-8 affected wing formation, we developed a genetic assay to test whether miPEP-8 acts through miR-8 using a previously validated miR-8 sponge, which titrates *miR-8* hence rescuing *miR-8*-induced phenotypes [29, 33]. Using this rescue assay, we asked whether the miR-8 sponge could also compensate the miPEP-8 induced phenotype. Co-expressing *miR-8* together with a miR sponge scramble (as a control and to maintain the number of UAS transgenes identical) using the MS1096 GAL4 driver led to wing size reduction. This phenotype was efficiently rescued by co-expressing *miR-8* with the effective miR-8 sponge (Fig. 5). In contrast, when miPEP-8 was co-expressed with the *miR-8* sponge, no compensation of the miPEP-8-induced wing reduction was observed. Therefore, this result strongly suggests that miPEP-8 acts in parallel of *miR-8*.

**Fig. 6:**
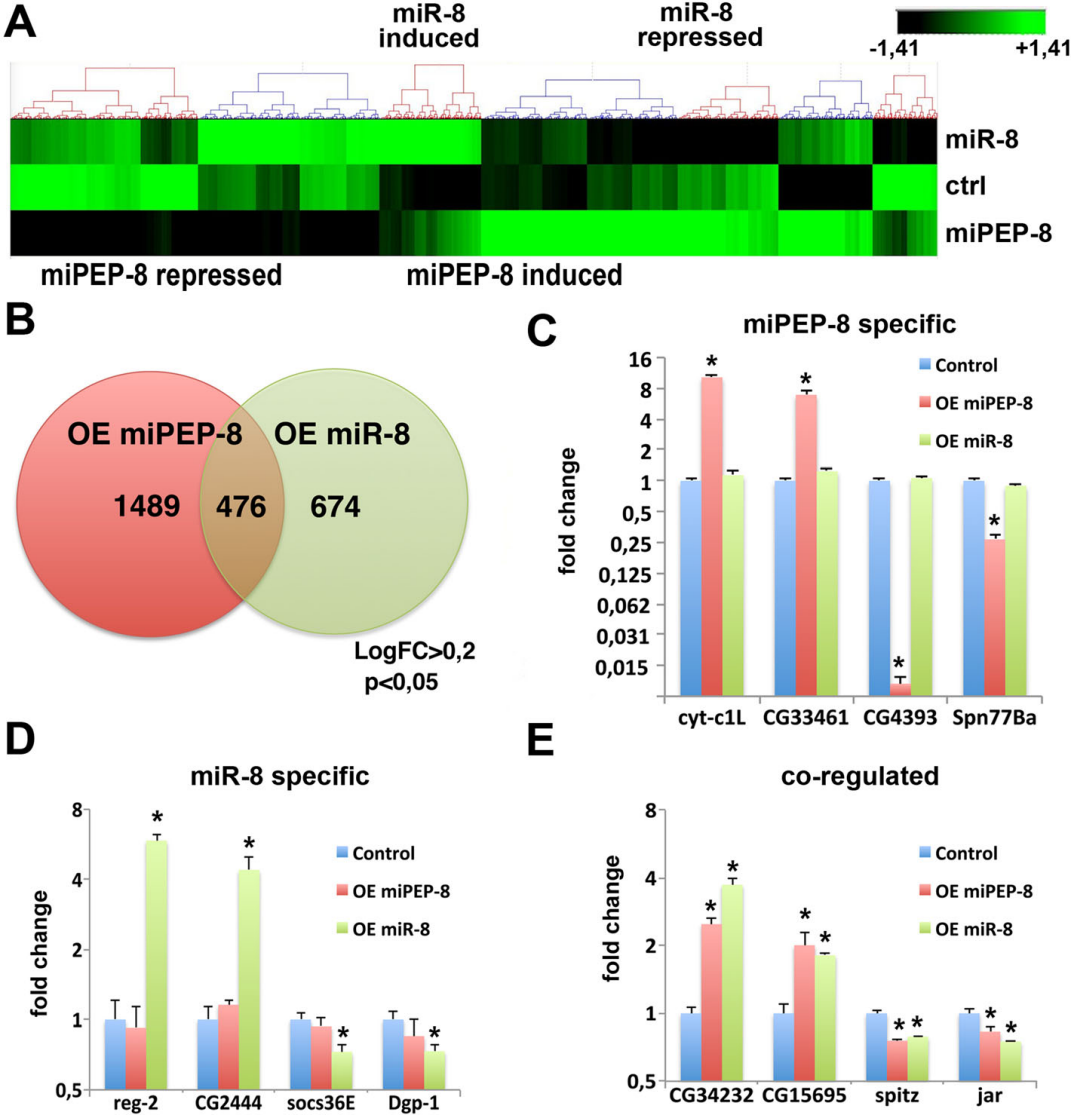
miR-8 and miPEP-8 control distinct set of genes. **a** Heatmap representing the RNA-seq results obtained from S2 cells over-expressing either *miR-8* or miPEP-8. Significant sets of genes are modulated in response to *mirR-8* or miPEP-8 over-expression, when compared to control transfected cells (ctrl). *N* = 5. **b** Venn diagram representing the *miR-8* versus miPEP-8 modulated genes. **c–e** Different subgroups are distinguished; miPEP-8 specific (**c**), *miR-8* specific (**d**) and co-regulated by miPEP-8 and *miR-8* (**e**)

We thus reasoned that the effect of miPEP-8 on wing development could be linked to the modulation of gene expression independent of *miR-8*. To identify these putative miPEP-8-regulated genes and to compare them with the *miR-8*-regulated transcriptome, we overexpressed *miR-8* or miPEP-8 in S2 cells and performed RNA-seq 48 h after transfection (Fig. 6; Additional file 3). Clearly, the transcriptomes appeared different (Fig. 6a; Additional file 1: Fig. S9). The assays performed on *miR-8* over-expressing cells successfully retrieved previously identified *miR-8* targets both at the RNA (Additional file 1: Fig. S9A and Additional file 2) and protein level (Additional file 1: Fig. S6), hence validating our experimental conditions. GO term enrichment identified biological pathways fitting with miR-8 activity such as “regulation of organism or cell growth and differentiation”, “wing development”, “apoptosis” and “regulation of actin cytoskeleton” (Additional file 1: Fig. S10). As for miPEP-8 controlled genes, strikingly, the majority of them were miPEP-8 specific (76%) (Fig. 6b, c) since only 24% appeared co-regulated (Fig. 6b, e). In both cases, we found activated and repressed genes (Fig. 6a, c–e). Remarkably, miPEP-8-modulated genes were frequently more strongly modulated than *miR-8*-modulated genes (Fig. 6c, d). Increasing the Fold change (FC > 1.5) led to a decrease of the number of genes but the respective proportions and conclusions remained unchanged (Additional file 1: Fig. S9B). Our analyses of GO term enrichment clearly identified shared functions for *miR-8* and miPEP-8 (Additional file 1: Fig. S10A and S11A; Additional file 4), some of which being related to wing morphogenesis (such as cell junction organization actin filament-based processes, epithelial cell morphogenesis, cell differentiation) or developmental processes (such as neurogenesis, cell migration, embryonic morphogenesis) (Additional file 4). However, *miR-8* and miPEP-8 also exhibit specific biological functions such as snRNA modification and leucine metabolic process for miR-8 or K48 linked ubiquitination and chromatin-mediated maintenance of transcription for miPEP-8 (Additional file 1: Fig. S10B and S11B).

Altogether, these experiments suggest that *miR-8* and miPEP-8 independently control similar biological processes, while regulating functions specific to one or the other.

## Discussion

In the present study, we investigated whether a small ORF present in *Drosophila pri-miR-8* was capable of producing a miPEP-8 and we propose that animal miPEPs are able to act in parallel of their corresponding miRs. Several studies performed in a broad range of organisms have revealed the prevalence of translated small/short open reading frames (smORFs/sORFs) [7, 44, 45, 52–57]. Although sORF peptides were initially identified as being encoded by unusual long non-coding RNAs, to date, it turns out that many classes of RNAs can produce these peptides. Therefore, sORF-encoded peptides (SEPs) are emerging as an unexplored reservoir of putative regulators. However, while a growing body of evidence further supports the importance of sORFs and associated peptides in development, physiology and diseases [8, 55, 58, 59], the number of SEPs that have been characterized so far still remains limited. Therefore, the current challenge resides in deciphering the full repertoire of their functions and molecular modes of action, an issue largely dependent on experimental approaches.

We show with several experimental data that a miPEP-8 is indeed produced from *Drosophila pri-miR-8*. First, we found a signal of ribosome binding in the *pri-miR* of several microRNAs and in particular, *miR-8*. Second, we show that the initiation codon of the miORF present within *pri-miR-8* is in a favorable translational context. Third, after having generated specific antibodies, we detected a peptide co-migrating with in vitro translated miPEP-8 in fly extracts. Fourth, forced expression or loss of function of this peptide led to a significant developmental phenotype in *Drosophila* and induced significant variations of cellular gene expression. Therefore, the poor conservation detected among *Drosophila* species indicates that this sORF-encoded peptide differs from the few conserved ones characterized so far and shed light on it by its recent invention.

Here, we tackled the question of whether the miPEP auto-regulatory function was identical to that of plants. While we did not detect any auto-regulatory loop (miPEP increasing the expression of its own *pri-miR* and miR), we observed that the action of miPEP-8 is uncoupled from *miR-8* regulation. On the one hand, our data suggest that this peptide could control similar developmental outcomes or developmental pathways and share the regulation of identical subsets of genes. In this context, we further analyzed whether we could detect a significant miPEP-8 activity in other *miR-8* developmental processes such as intestinal stem cell differentiation [27] and eye morphogenesis [37] (data not shown). However, no significant activity was detected, suggesting that, in the experimental conditions tested, miPEP-8 does not act in all *miR-8* developmental pathways. Such an example was observed in S2 cells in which *miR-8* is expressed at detectable levels whereas endogenous miPEP-8 is not. On the other hand, we reveal that miPEP-8 likely has a regulatory function all of its own, independently of *miR-8*. Indeed, our RNA-seq data indicates that miPEP-8 regulates specific genes and biological processes (i.e. independent of *miR-8* activity). This also occurs in vivo since few candidates of the top list of miPEP-8 specific regulated genes identified in S2 cells are also modulated in miPEP-8 loss of function in adult flies (Additional file 1: Fig. S12). Future loss of function and expression pattern analyses throughout development should bring further insight into miPEP-8-specific regulatory functions.

Is the uncoupling of miPEP activity from miR regulation a general feature of animal miR genes? The study performed here suggests that the mechanisms involved in animals might be different from the miPEP auto-regulatory mechanism observed in plants. As such, a recent study on human miR155 revealed an activity for a miPEP155 that is not correlated to miR155 control [20] and on human miR34 where a miPEP133 mitochondrial function impinging on p53 activity was reported [21]. In light of these results, of course, we cannot affirm that the mechanisms described here are common to all *Drosophila miR* genes. It remains possible that some of them might be auto-regulated by their miPEPs as described in plants. In addition, since ribosome occupancy were not found in all *Drosophila* *miR* genes, it remains possible that some *pri-miRs* are unable to produce miPEPs. Therefore, additional studies will be required to determine whether miPEP-dependent *pri-miR* auto-regulation is specific or widespread among *miR* genes.

Is the *pri-miR* coding capacity conserved throughout the animal kingdom? In a search for non-coding RNAs able to express sORF-encoded peptides, Razooki and co-workers found that human *miR-22* host gene (*pri-miR-22*) produces a potential miPEP-22 that is induced during viral infection [19]. sORFs have also recently been identified in *miR-200a* and *miR-200b pri-miRs*, the human orthologues of the *Drosophila miR-8*. Like *miR-200a* and *miR-200b*, miPEP-200a and b over-expression in prostate cancer cells inhibits migration of these cells by regulating the vimentin-mediated pathway, suggesting that the miPEP-coding function of *pri-miRs* is present in humans [18]. Accordingly, most recently, micropeptides encoded by MIR155HG and MIR34HG were described to be involved in autoimmune inflammation by controlling antigen presentation [20] and mitochondrial function respectively [21] via their interaction with different HSP proteins. It is interesting to note however, that these miPEPs appear to be involved in infections/pathologic-conditions, hence suggesting that revealing miPEP function might be largely dependent on the biological context.

Ribosome-associated lncRNAs has been considered to constitute a hallmark of protein translation. Here we found a signal of ribosome binding in the *pri-miR* of several Drosophila *miRs* genes. Furthermore, we showed that the initiation codon of the miORF present within *pri-mR-8* is in a favorable translational context. Indeed, after having generated specific antibodies, we detected a peptide co-migrating with in vitro translated miPEP-8 in fly extracts. However, an alternative possibility proposed by others is that ribosome marks illustrate a mechanism for cellular control of lncRNA levels through ribosome degradation-promoting activity [57, 60]. It will be of interest to investigate further whether the short ORFs present in *pri-miRs* are able to influence their regulation by controlling their stability and degradation as it has been shown for other coding genes. Finally, the molecules that give rise to miR-8/miPEP-8 are probably not the same ones since Drosha processing would separate the ORF from the poly(A) tail and thereby cause rapid decapping and degradation of the ORF-containing fragment. In light with these considerations, it is difficult to conclude on a pervasive coding capacity of *pri-miRs* in *Drosophila*. Future work will determine both in plants and animals whether all of them are sources of miPEPs and to what extent their auto-regulatory capacity and/or modes of action are diverse and specific.

## Conclusion

Many studies performed recently have led to functional characterization of a handful of additional SEPs in the plant and animal kingdom. Illustrating the diversity of functions of these new players, these SEPs were identified from different sources of RNAs and play different roles [9, 61, 62]. Among these, contrasting with their initial definition as non-coding, pioneer works in plants showed that even precursors transcript of miRs produces SEPs involved in an autoregulatory feedback loop. By addressing the conservation of this mechanism in animals, our findings combined with others confirm that miR-encoded genes probably represent evolutionary conserved bi-functional RNAs carrying coding and non-coding functions. However, contrasting with the mechanism described in plants, our data shed light on the diverse functions fulfilled by microRNA-encoded-peptides despite their poor conservation among *Drosophila species*.

## Methods

### Fly strains and genetics

*Drosophila* flies were maintained on standard cornmeal-yeast medium (Dutscher). Experiments were performed at 25 °C when *miR-8* GAL4 (NP5247) was used as driver. For the experiments of wing phenotype of flies expressing transgenes under the control of MS1096 Gal4, crosses were placed at 28 °C. UAS-*pri-miR-*8 and UAS-miPEP-8 transgenic lines were inserted in attP86F site through PhiC31-mediated integration. Injections were performed by Bestgene Inc. (USA). Generating pri-miR-8 fly founder line: pri-miR-8 fly founder line was designed and generated by inDroso Functional Genomics (Rennes, France) using CRISPR/Cas9. The pri-miR-8 fly founder line was generated by excising from position 16830745 to 16831521 on Chromosome 2R arm and replacing it by an attP::loxP::3xP3-dsRED::loxP cassette (Additional file 1: Fig. S8). The two following guide RNA sequences were used to cut on either side of the *pri-miR-8*: CACATATG|CAACGGAAAGAG and GTTGGTGG|TACTGAAGGTTA. The edited region was verified by sequencing. The two pri-miR-8 constructs in pattB were inserted at the Δ*miR-8* created P site. Three independent transformants were used for analyses. The same strategy was used to generate the driver *miR-8* GAL4, Δ*miR-8.* The miPEP-8 alternative form creating a premature stop codon in miPEP-8 was derived from the DGRP-859 line, outcrossed into white recipient flies and kept over CyO. Experiments are the sum of at least 3 independent crosses. *n* indicates the number of individuals analyzed. For wing measurements, young flies (2–5 days) of the appropriate genotypes were stored in ethanol. For analysis of wings, females adult wings were removed in wash buffer (PBS and 0.1% Triton X-100) and mounted on a slide in 80% glycerol in PBS as described [63]. Wings or wing discs images were acquired on a Zeiss Axiozoom stereomicroscope. Measurements of wing size were performed using ImageJ software*.*

### Molecular methods

For cloning procedure, miPEP-8, miR-8 or pri-miR-8 plasmids were constructed from PCR amplification of genomic DNA, gene synthesis or by RNA reverse transcription from S2 cells or adult *Drosophila* RNA and cloned in pUAS-attB vector constructs using the In-fusion HD cloning kit (Takarabio) according to the manufacturer specification. All constructs were verified by sequencing. For quantitative PCR experiments, total RNA was isolated from young adult fruit flies (2–5 days) or S2 cells using TRI Reagent (Sigma) according to the manufacturer specifications, followed by RQ1DNase treatment (promega) according the manufacturer specifications. The cDNA template was synthesized using SuperScript III (Invitrogen) with oligo-dT18 as anchor primers. Quantitative real-time PCR was performed on the LightCycler 480 Instument II (Roche Life Science) using LightCycler480 SYBR GREEN I master (Roche Life Science). The mRNA abundance of the examined genes was estimated by qPCR. For the endogenous *pri-miR* or coding genes, RP49 and tubulin genes were used as reference genes and used for normalization. For quantifying mature miRNA, stem loop PCR conditions were set up and the small RNAs U14 and Sno442 were used as reference. Datas presented are the same whatever the reference gene used. When the S2 cells are transiently transfected, the co-transfected pActin-GAL4 vector (Addgene # 24344) was used to monitor transfection efficiency. Typically, each experiment presented was performed with four replicates processed independently and was repeated in time at least three times. All experiments were taken into account and “*n*” indicate the total number of biological replicates used for analyses. Primers used in the qPCR are listed in the Additional file 1: Fig. S13.

### RNA analysis

For Rib-seq analyses, Dmel primiR three frames translations have been performed with transeq (Emboss suite 6.6.0). A homemade script written in Perl was generated to compare the resulting translated peptides to the ribo-seq sORF-encoded peptides described in [4, 46, 47]. RNA-seq was processed by genewiz (Germany). Each dataset contains five independent biological replicates of control *miR-8* and miPEP-8 over-expressing S2 cells RNA-seq. The reads were subjected to standard quality control (QC) and filtered according to the following parameters: (1) trimming and cleaning reads that aligned to primers and/or adaptors, (2) reads with over 50% of low-quality bases (quality value ≤15) in one read and (3) reads with over 10% unknown bases (N bases). We used Trimmomatic software (v0.36) to remove primers and bad quality reads. After filtering, we removed short reads (parameters were used with default values). Gene and PSI lists for each dataset were compared to identify common events between them. For RNAseq analysis, htseq-count files were analyzed using the version 3.24.3 of package EdgeR [64], in order to normalize raw counts by “trimmed mean of M-values” (TMM), and test differential expression using the negative binomial distribution. RNA-seq analysis: raw *p* values were adjusted with the Benjamini–Hochberg procedure to control the false discovery rate (FDR). A gene was declared differentially expressed if it is adjusted *p* value ≤0.05. Heat map parameters applied: row-by-row normalization by standardization (mean and standard deviation). GO term analysis was performed with PANTHER (http://pantherdb.org/) [65]. Sashimi plots were created with IGV (Integrative Genomics Viewer, https://igv.org/) and GWIPS-viz [42]. Statistical analyses were performed using the version 3.5.2 of R software and Bioconductor packages. For QPCR analysis, the version 1.3-1 of package Agricolae was used.

### Cell culture and western blot and luciferase assays

*Drosophila* S2 cells were maintained in Schneider’s medium (Invitrogen) supplemented with 10% fetal bovine serum (Sigma), 50 U/ml penicillin and 50 μg/ml streptomycin (Invitrogen) at 25 °C. For western blot experiments, miPEPs sequences cloned into pF25A ICE T7 Flexi vector were expressed in vitro using TnT® T7 Insect Cell Extract Protein Expression System (Promega). For cells extracts and *Drosophila* extracts, we directly freeze them in nitrogen just before western blot. Proteins were prepared in Laemli buffer (63 mM Tris HCl pH 7.5, 2% SDS, 5% 2-mercaptoethanol) and run on SDS-PAGE according to [13]. Primary antibodies used for western were as follows: rabbit anti-miPEP-8 were raised against the sequence KQSDKQNSKERKKNTQI (generated and affinity purified by Agro-bio, France), mouse anti-GAPDH (ThermoFisher AM4300), rabbit anti-Sra-1 (1/1000, provided by A. Giangrande, IGBMC CNRS, France), mouse anti-peanut (1/100, DSHB, USA) and rabbit anti-ABP-1 (1:250, provided by Michael Kessels Jena University Hospital, Germany). HRP-conjugated secondary antibodies are from Santa Cruz Biotechnology (1/10000 sc-516102). For luciferase assays, in each experiment, S2 cells were transfected in quadruplicate, in 24-wells plates (700,000 cells/well) using FuGene HD transfection Reagent (Promega). Experiments were repeated timely independently at least 3 times. After 48 h of transfection, cells were washed with PBS and lysed with 100 μL passive lysis buffer (Dual Luciferase Reporter Assay System, Promega). Firefly luciferase (FL) and Renilla luciferase (RL) activities were then quantified with Dual Luciferase Reporter Assay (Promega) using 50 μl of reagents/well and measure using a Greiner luminometer instrument.

### Statistical analyses

Statistical analyses were performed using GraphPad Prism and illustrated as follow: *p value< 0.05, **p value< 0.01, ***p value< 0.001 and ****p value< 0.0001. In all experiments, results represent mean ± s.e.m. *n* represents the number of biological independent replicates. Normality test was first performed using D’Agostino Pearson test. If the distribution is Gaussian and in order to detect a global difference between all groups, one-way ANOVA was performed using one-way analyses of variances followed by Bartlett’s test for equal variance and Bonferroni’s multiple comparison tests. In other cases, when variance or sample sizes are not equal, non-parametric analyses were performed using Kruskal-Wallis test to detect a global difference between all groups followed by comparisons between two groups performed using adjustments for multiple comparisons. When only two groups were compared, a Mann-Whitney test was performed.

## Supplementary Information

**Additional file 1.** Supplementary Figures S1 to S13.

**Additional file 2.** list of sORF peptides defined from Rib-seq analyses.

**Additional file 3.** Normalized RNA-seq values from S2 cells.

**Additional file 4.** Protein ANalysis THrough Evolutionary Relationships (PANTHER) Overrepresentation Test.

**Additional file 5.** Uncropped Western blots.

**Additional file 6.** Review history.

## Data Availability

The RNAseq datasets generated during the current study are available in the NCBI Gene Expression Omnibus (GEO) repository under the accession number BioProject ID PRJNA645280 [66] [https://www.ncbi.nlm.nih.gov/Traces/study/?acc=PRJNA645280&o=acc_s%3Aa].
