# Supplementary material for: Drosophila primary microRNA-8 encodes a microRNA-encoded peptide acting in parallel of miR-8
Source: Genome Biol. 2021 Apr 23;22:118. doi: 10.1186/s13059-021-02345-8 (PMC8063413; doi:10.1186/s13059-021-02345-8)
Supplement: Supplementary file 5 — Additional file 5. Uncropped Western blots. [file 13059_2021_2345_MOESM5_ESM.pdf]

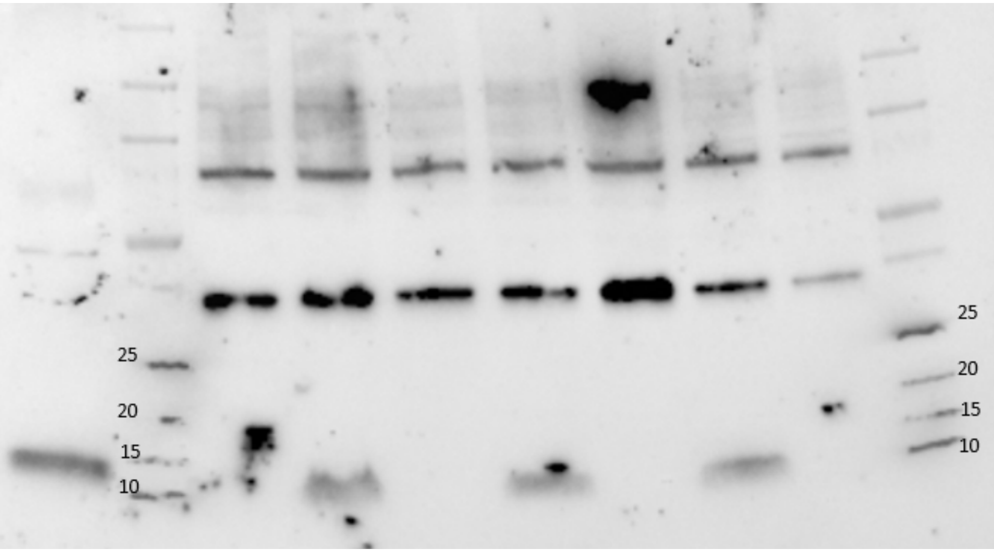

TnT ATG1 Marqueur NT miPEP8koz miPEP8kozMT Phiphi Phiphi MT milong milong MT Marqueur

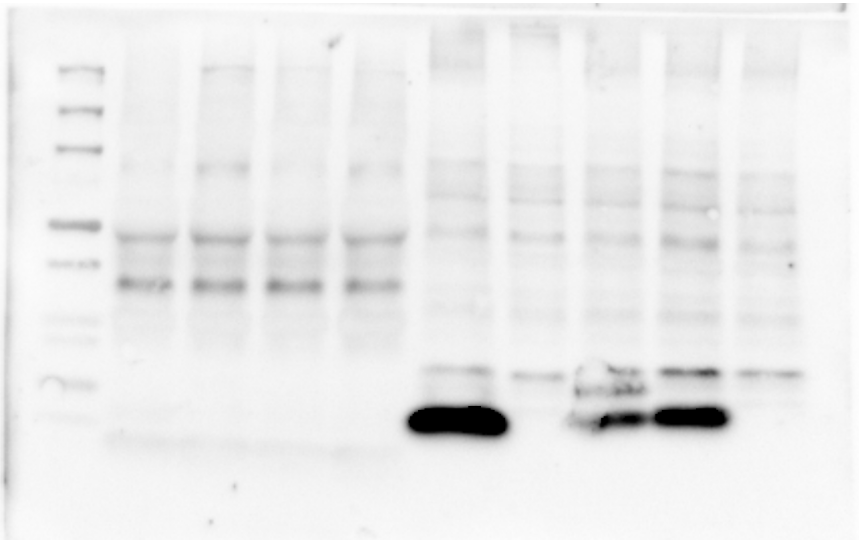

ATG1 - CTG ATG1 ATG2

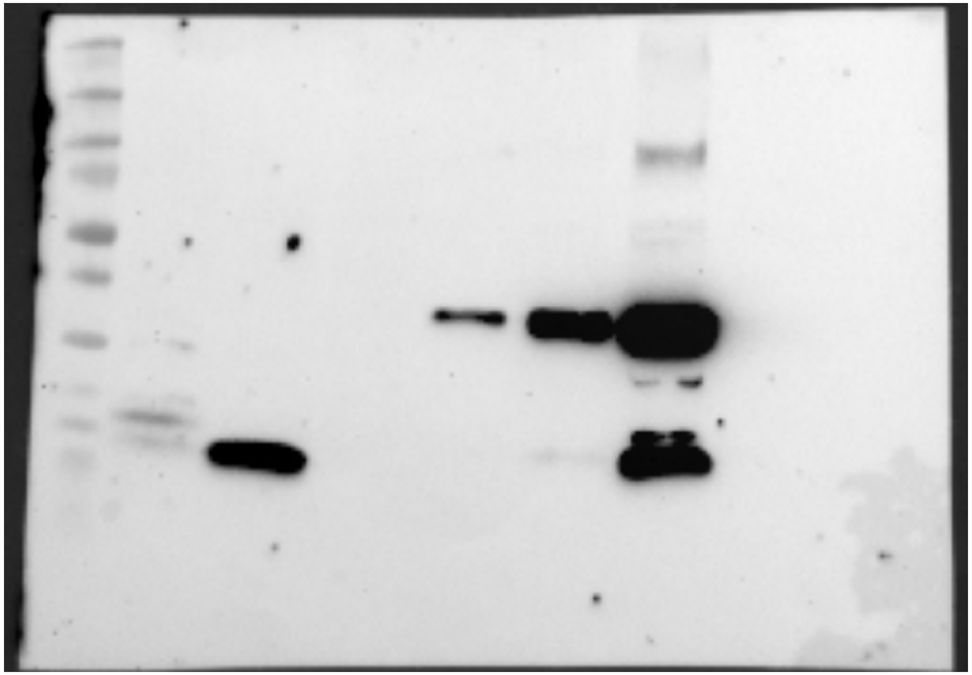

CTG ATG1 - 1µl 3µl 10µl D.mel (white)

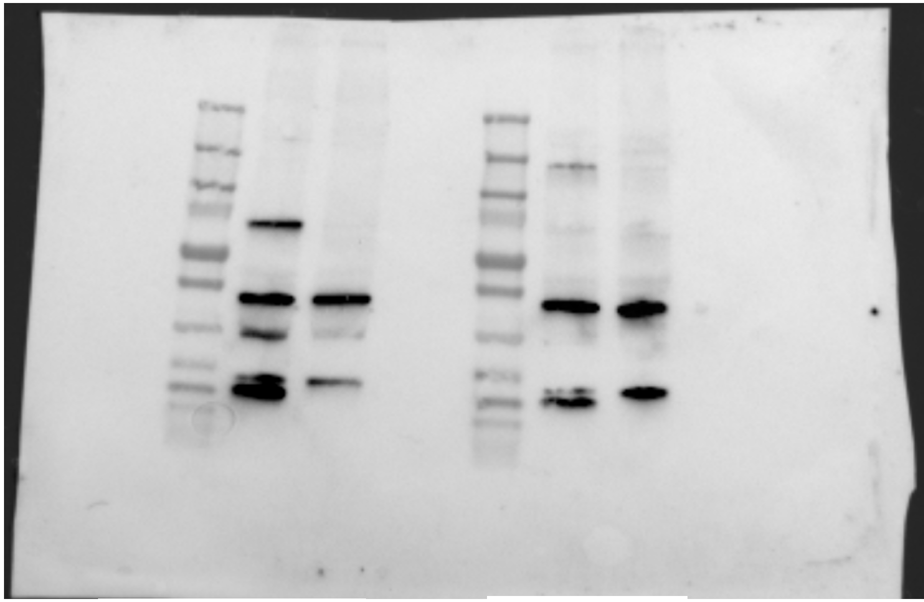

5µl white Δ2 white Δ2

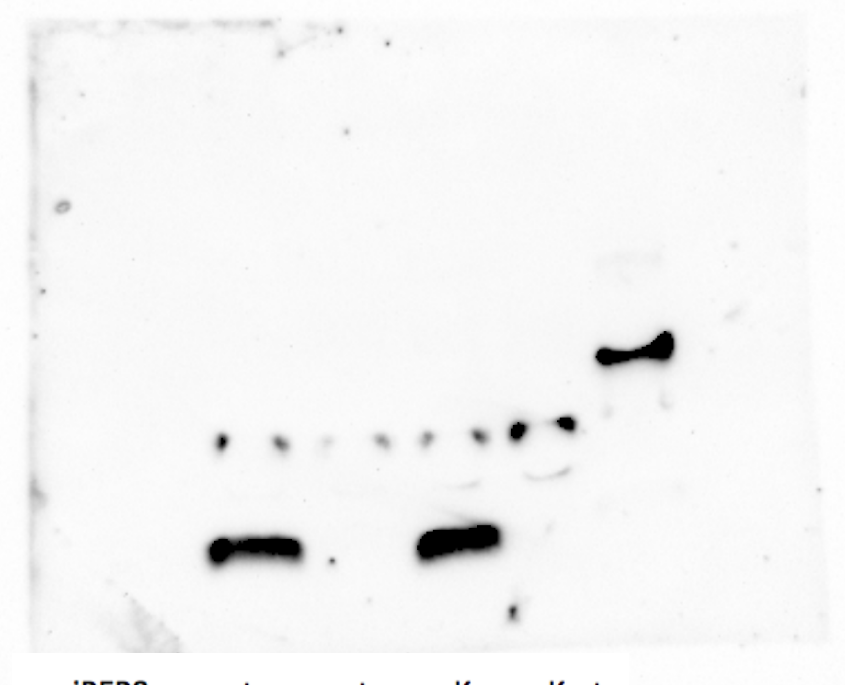

miPEP8 wt mt K Kmt

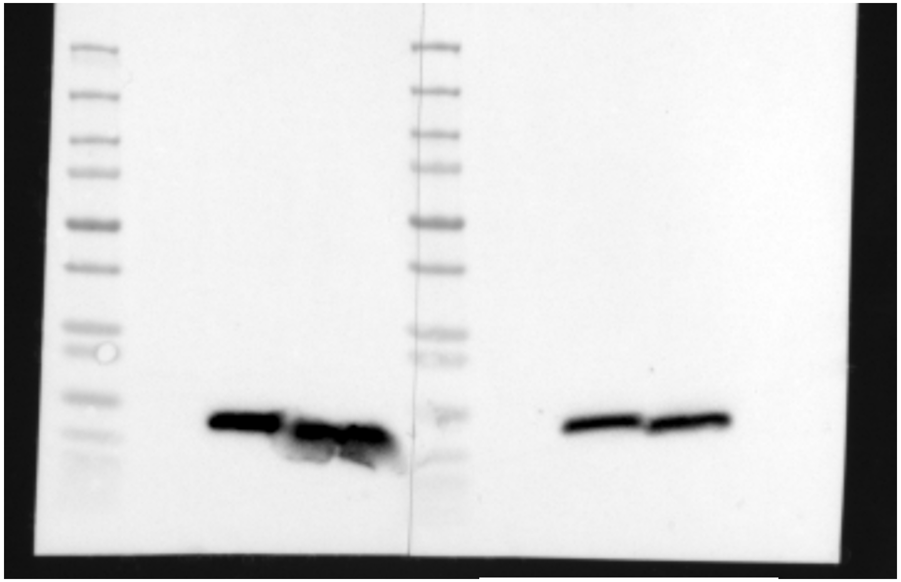

ctrl Atg1HA Atg1 ctrl Atg1HA Atg1

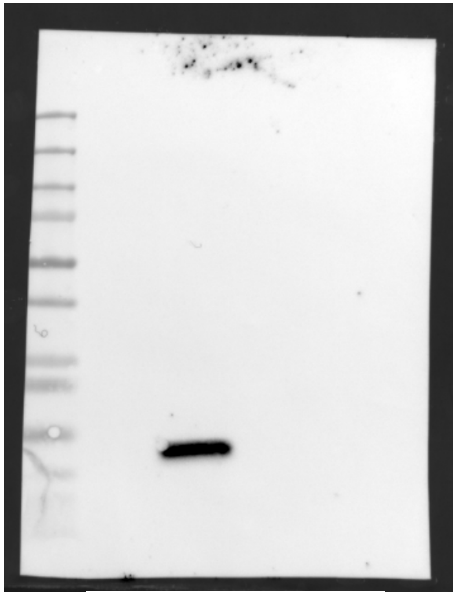

ctrl Atg1HA Atg1

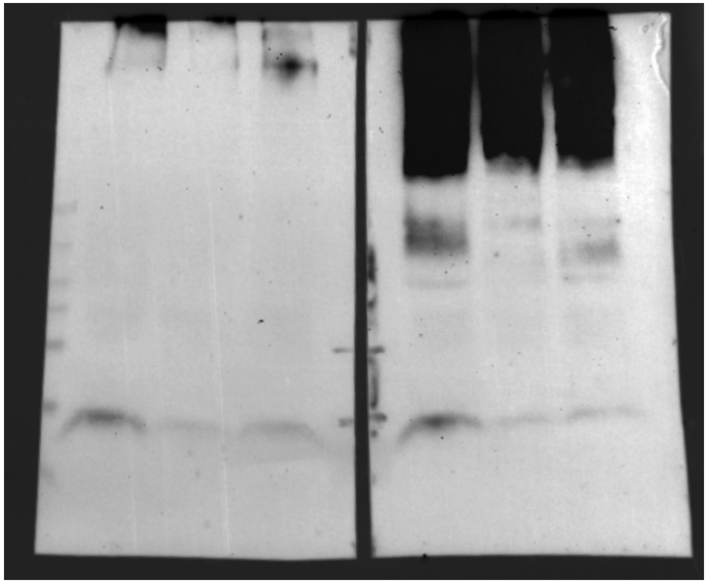

OE8 OE8mt ctrl OE8 OE8mt ctrl

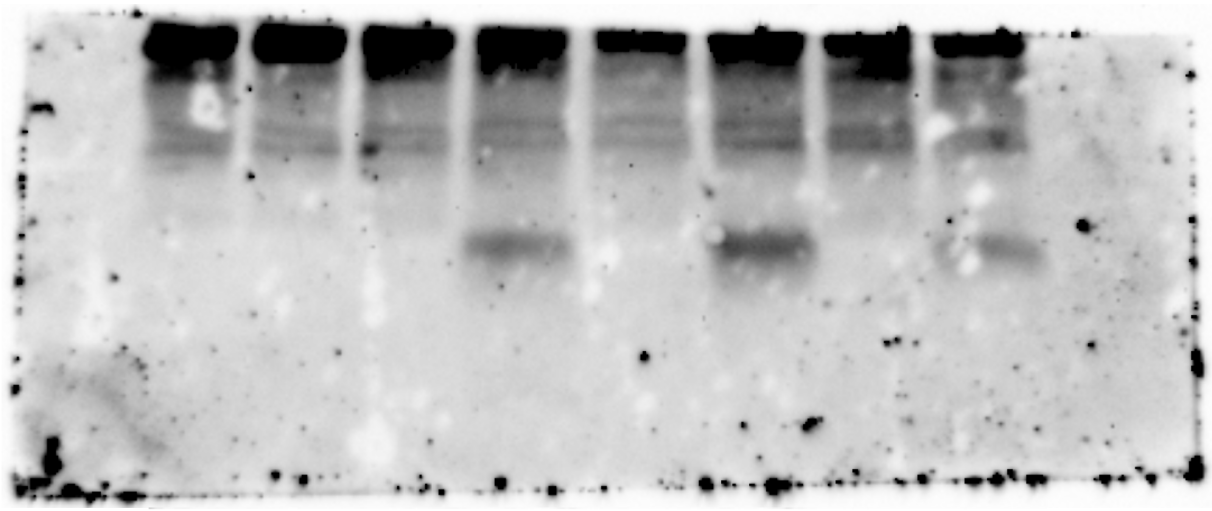

- OE8_1 _6,3 _5,2 - OE8_2 _6,6 _5,1

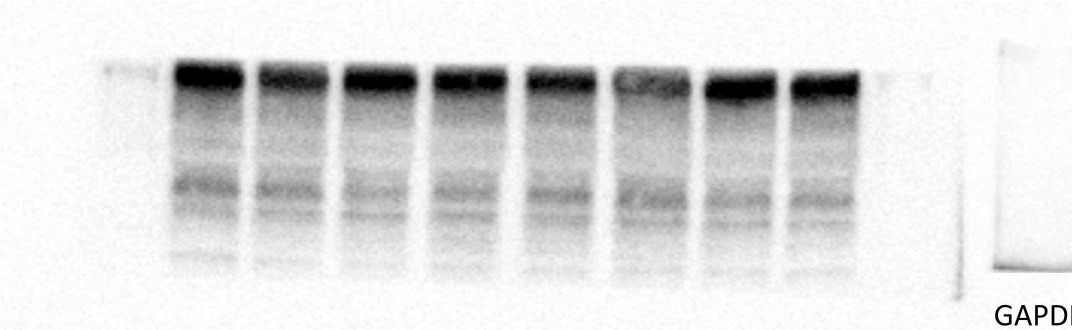

ABP1 1 2 3 4 5 6 7 8

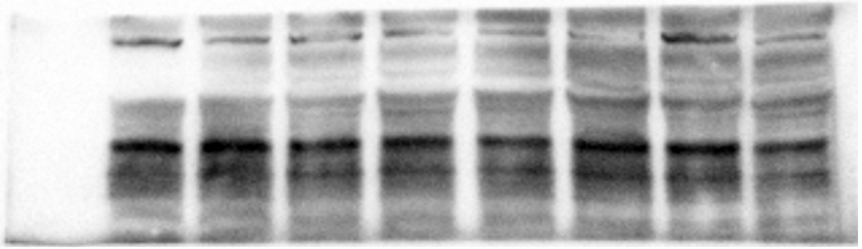

GAPDH 1 2 3 4 5 6 7 8

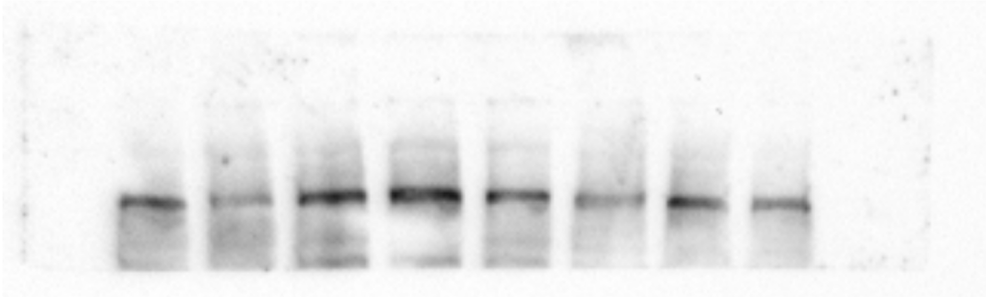

lanes 1,5 NT
lanes 2,6 OE miR8
lanes 3,7 OE miPEP8
lanes 4, 8 OE miPEP8m

Sra-1
