# Supplementary material for: Drosophila primary microRNA-8 encodes a microRNA-encoded peptide acting in parallel of miR-8
Source: Genome Biol. 2021 Apr 23;22:118. doi: 10.1186/s13059-021-02345-8 (PMC8063413; doi:10.1186/s13059-021-02345-8)
Supplement: Supplementary file 6 — Additional file 6. Review history. [file 13059_2021_2345_MOESM6_ESM.docx]

Review History

**First round of review**

**Reviewer 1**

**Are you able to assess all statistics in the manuscript, including the appropriateness of statistical tests used?** Yes, and I have assessed the statistics in my report.

**Comments to author:**

Based on previous studies that reported the expression of regulatory peptides encoded in primary transcripts from miRNAs, here the authors first perform a survey of ribosome profiling data and confirm the presence of translating ribosomes on pri-mi-RNAs in drosophila, then further investigate a micropeptide encoded in pri-miR-8, miPEP-8. These investigations include raising specific antibodies against miPEP-8, reporting the functional consequences of overexpressed miPEP-8 and addressing the potential biological coupling between miPEP-8 and miR-8. They conclude that Drosophila miPEP-8 is not involved in a positive auto-regulatory feedback loop as observed for miPEPs described in plants.

The analyses performed in this study are very well performed, including the sstatistical analyses, and the biological function of OVEREXPRESSED miPEP-8 in terms of development and gene expression is very convincing. However, two major issues must be addressed to complete this study (see below). As it stands, the study is too preliminary to convince the reader of the endogenous expression of miPEP-8 and of its physiological function. This is all the more important as the results suggest that the mechanism of action of miPEP-8 is different from the mechanism associated with miPEPs in plants. Such a conclusion is very important and should be based on the indisputable endogenous expression of miPEP-8.

MAJOR ISSUES

1. Figure 1D.

In the absence of KO, the demonstration that the band detected at the expected size in vivo (right blot) genuinely represents endogenous miPEP-8 is incomplete. An alternative would be to perform a knockdown experiment in a cell line endogenously expressing miPEP-8. Unfortunately, endogenous miPEP-8 is not detected in S2 cells (ctrl lane, middle blot; and ctrl lane right blot). The author should perform a KO or KD experiment to convincingly demonstrate that miPEP-8 is endogenously expressed.

The authors should comment on the fact that miPEP-8 is not expressed in S2 cells. Is miR-8 expressed in these cells?

2. The biological activity reported in the manuscript (e.g. wing defects) relies on overexpression experiments only. miPEP-8 loss of function mutant flies would allow the authors to confirm some of the biological findings.

OTHER ISSUE

In vivo, in addition to a band at the expected size for miPEP-8, the anti-miPEP-8 antibodies also detect a 30 kDa band. Actually, the signal for this band is way much stronger that the signal for miPEP-8. One simple explanation is that of a nonspecific signal. However, it is possible that it also represents miPEP-8 (i.e. trimeric form? PTM modifications?). Could the authors elaborate on that?

**Reviewer 2**

**Are you able to assess all statistics in the manuscript, including the appropriateness of statistical tests used?** Yes, and I have assessed the statistics in my report.

**Comments to author:**

Inspired by reports that some plant pri-miRNAs code for short peptides that appear to feed back on the expression of the miRNA, the authors look for a similar phenomenon in flies. They use ribosome profiling data to find Drosophila pri-miRNAs with evidence of miPEP translation, and follow-up on one of these candidates, a peptide produced from the D. melanogaster pri-miR-8. These follow-up experiments provide evidence that a peptide is produced and that this peptide can alter development and gene expression when over-expressed.

Major concerns:

1. The interest in this paper rests on the claim that miPEP-8 has a biological function. For this purpose, the results of over-expression studies are a step in the right direction, but not convincing one their own. To convincingly show biological function, the authors need to also perform loss-of-function function experiments and observe a loss-of-function phenotype. The authors already seem to have a fly line that lacks the peptide (lines 188-191), but if not, with CRISPR, it is straight-forward to knock out the peptide and look for changes in phenotype and mRNA levels.

2. In the absence of any loss-of-function data, it would be helpful to know if the peptide is conserved any more than expected by chance. Unfortunately, the analysis of peptide conservation provided by the authors is very weak and gives the mistaken impression that the peptide CDS is under negative selection. The analysis of SNPs (lines 178-184) does not give a statistically significant result, and an analysis of conservation among insect or Drosophila whole-genome alignments is missing. Instead, the authors cherry pick the 5 additional Drosophila species in which ATG1 happens to be conserved and show an alignment of the predicted peptides from these species, failing to mention that the peptide is not conserved in any of the other sequenced Drosophila species. Moreover, when one does look at the whole-genome alignments at the UCSC browser, examining the small clade that has the predicted peptide, the pattern of conservation is not what would be expected for a functional peptide. For example, the coding nucleotides are no more conserved than flanking nucleotides, and there is no sign of the triplet periodicity, with more variable silent positions, expected for a peptide CDS under negative selection.

Minor concerns:

Lines 31-32. This sentence makes it sound like lncRNAs generally produce functional small peptides. This is has not been shown to be true. Many lncRNAs that get into the cytoplasm are translated to produce short peptdies, and in some cases these peptides are stable, and in a few of these cases the peptides have been shown to be functional. However, function has been shown for only a small fraction of the peptides deriving from lncRNAs. Indeed, the conventional view is that once function has been shown, the transcript is considered an mRNA that was misannotated as a lncRNA. By this convention, no authentic lncRNAs (which are noncoding by definition) code for a functional peptide. Similarly, lines 75-76 might be interpreted to be saying that myriads of lncRNA ORFs are translated into functional peptides, which was not shown in refs 4-7.

Line 37. The logic of searching for the conservation of this regulatory feedback loop in plants and animals is flawed. For this logic to make sense, the miRNA pathway itself would have to be conserved from plants to animals. However, the current view is that the miRNA pathways of plants and animals emerged independently, from a common ancestor that had RNAi but not miRNAs.

Line 57. Might be good to say it has been more than 20 years, or to specify the genome that was annotated. Yeast was sequence in 1996.

Line 91-92. This sentence dismisses the miPEPs reported in humans as not necessarily involved in miRNA expression. However, since miPEP-8 itself does not influence the expression of miR-8, it would seem that the reported activities of the human miPEPs should be described in the introduction and not so readily ignored.

Line 193. The use of "functional ORF" might be misinterpreted to be implying that the peptide is known to be functional. The term "translated ORF" would avoid this confusion.

Fig. 5. The RNA-seq analysis seems to be focusing on very small changes with the color-key maxing out at 1.41-fold changes. Also, the analysis of statistical significance is not clear. Which changes are significant (after multiple-hypothesis adjustment)? What is the FDR?

Discussion: It would be useful to mention that the molecules that give rise to miR-8 are probably not the same ones that are translated to produce miPEP-8, since Drosha processing would separate the ORF from the poly(A) tail and thereby cause rapid decapping and degradation of the ORF-containing fragment.

**Authors Response**

**Point-by-point responses to the reviewers’ comments:**

Reviewer #1: Based on previous studies that reported the expression of regulatory peptides encoded in primary transcripts from miRNAs, here the authors first perform a survey of ribosome profiling data and confirm the presence of translating ribosomes on pri-mi-RNAs in drosophila, then further investigate a micropeptide encoded in pri-miR-8, miPEP-8. These investigations include raising specific antibodies against miPEP-8, reporting the functional consequences of overexpressed miPEP-8 and addressing the potential biological coupling between miPEP-8 and miR-8. They conclude that Drosophila miPEP-8 is not involved in a positive auto-regulatory feedback loop as observed for miPEPs described in plants.
The analyses performed in this study are very well performed, including the sstatistical analyses, and the biological function of OVEREXPRESSED miPEP-8 in terms of development and gene expression is very convincing. However, two major issues must be addressed to complete this study (see below). As it stands, the study is too preliminary to convince the reader of the endogenous expression of miPEP-8 and of its physiological function. This is all the more important as the results suggest that the mechanism of action of miPEP-8 is different from the mechanism associated with miPEPs in plants. Such a conclusion is very important and should be based on the indisputable endogenous expression of miPEP-8.

MAJOR ISSUES
1. Figure 1D.
In the absence of KO, the demonstration that the band detected at the expected size in vivo (right blot) genuinely represents endogenous miPEP-8 is incomplete. An alternative would be to perform a knockdown experiment in a cell line endogenously expressing miPEP-8. Unfortunately, endogenous miPEP-8 is not detected in S2 cells (ctrl lane, middle blot; and ctrl lane right blot). The author should perform a KO or KD experiment to convincingly demonstrate that miPEP-8 is endogenously expressed.

A western blot in miR-8KO flies is now provided Figure 1. We show panel D that the miPEP8 is not expressed in null mutant flies.

The authors should comment on the fact that miPEP-8 is not expressed in S2 cells. Is miR-8 expressed in these cells?

*miR-8* is expressed in these cells. According to the Western Blot experiments Figure 1 panel D, it seems that the miPEP8 is not expressed at all or only at very low undetectable levels.

2. The biological activity reported in the manuscript (e.g. wing defects) relies on overexpression experiments only. miPEP-8 loss of function mutant flies would allow the authors to confirm some of the biological findings.

We tried several times to obtain specific miPEP8 editing at *miR-8* locus, but unsuccessfully. We developed a strategy by knock out/ knock in to compare the efficiency of pri-miR-8 with the miPEP-8 mutated version. miPEP-8 Loss of function on wings is now provided Figure 4 and show a wing size phenotype.

OTHER ISSUE
In vivo, in addition to a band at the expected size for miPEP-8, the anti-miPEP-8 antibodies also detect a 30 kDa band. Actually, the signal for this band is way much stronger that the signal for miPEP-8. One simple explanation is that of a nonspecific signal. However, it is possible that it also represents miPEP-8 (i.e. trimeric form? PTM modifications?). Could the authors elaborate on that?

The western blot experiment performed on miR-8 Δ2 deficiency flies revealed non-specific bands as well as additional specific bands (representing possibly multimeric forms or PTM modifications). This has been precised in the figure legend of Figure 1 lane 627 p22.

Reviewer #2: 
Inspired by reports that some plant pri-miRNAs code for short peptides that appear to feed back on the expression of the miRNA, the authors look for a similar phenomenon in flies.  They use ribosome profiling data to find Drosophila pri-miRNAs with evidence of miPEP translation, and follow-up on one of these candidates, a peptide produced from the D. melanogaster pri-miR-8. These follow-up experiments provide evidence that a peptide is produced and that this peptide can alter development and gene expression when over-expressed.  

Major concerns:

1. The interest in this paper rests on the claim that miPEP-8 has a biological function. For this purpose, the results of over-expression studies are a step in the right direction, but not convincing one their own. To convincingly show biological function, the authors need to also perform loss-of-function function experiments and observe a loss-of-function phenotype. The authors already seem to have a fly line that lacks the peptide (lines 188-191), but if not, with CRISPR, it is straight-forward to knock out the peptide and look for changes in phenotype and mRNA levels.

The reviewer is referring to the polymorphisms found in databases or RNA-seq analyses. Unfortunately, we did not have the chance to isolate such a fly stock.
We tried several times (during 4 years) to obtain by CRISPR a specific miPEP-8 edited line at the miR-8 locus, even by requesting help from specialized companies. It was not possible to get this edited line. By contrast, as control, it was possible from the first attempt to delete the entire miR-8 locus.

We used these lines to develop an alternative strategy by knock in to re-express the pri-miR-8 in *miR-8* mutant background using pri-miR-8 wt or pri-miR-8 miPEP8 mutated constructs. miPEP-8 LOF on wings is now provided as well as *miR-8* RNA levels Figure 4.

2. In the absence of any loss-of-function data, it would be helpful to know if the peptide is conserved any more than expected by chance. Unfortunately, the analysis of peptide conservation provided by the authors is very weak and gives the mistaken impression that the peptide CDS is under negative selection.  The analysis of SNPs (lines 178-184) does not give a statistically significant result, and an analysis of conservation among insect or Drosophila whole-genome alignments is missing.  Instead, the authors cherry pick the 5 additional Drosophila species in which ATG1 happens to be conserved and show an alignment of the predicted peptides from these species, failing to mention that the peptide is not conserved in any of the other sequenced Drosophila species.  Moreover, when one does look at the whole-genome alignments at the UCSC browser, examining the small clade that has the predicted peptide, the pattern of conservation is not what would be expected for a functional
peptide.  For example, the coding nucleotides are no more conserved than flanking nucleotides, and there is no sign of the triplet periodicity, with more variable silent positions, expected for a peptide CDS under negative selection.

We are now providing the requested loss-of-function data.

Considering the evolutionary conservation.

- we previously indicated in the text that the miPEP sequence was poorly conserved… We thought that it was most important to present the conserved sequences. We now precise this in the figure legend p22 and in the result section p7 lane 198. We apologize for this oversight.

- We modify the text of the result section (lane 186-188 p7) to present these data as a study and not evidences of miPEP8 existence.

It is true that conservation of ORFs across species can help at identifying functional small proteins that have been preserved by natural selection. However, only few of them have been identified through this way. JP Couso & Patraquim (2017) have proposed in their review that smORF peptides represent very young and rapidly evolving sequences. For example, the sarcolamban (Scl) family of short CDSs show low amino acid sequence conservation, but their structure and molecular function are conserved. One-half of canonical ORFs are conserved across the Kingdom, whereas short CDSs display significant conservation at the Class level. J Ruiz-Orera and M. Mar Albà (2019) also precised in their review that recently evolved de novo proteins are small and poorly conserved across species; therefore, many of them are likely to be missing from the current gene annotations, or being still annotated as noncoding loci.

We believe that the use of experimental methods to identify translated ORFs combined with this analysis represents one important aspect of our study and therefore help to close this gap to improve our understanding of the impact of *de novo* peptide/proteins in evolutionary innovation.

Minor concerns:

Lines 31-32. This sentence makes it sound like lncRNAs generally produce functional small peptides. This is has not been shown to be true. Many lncRNAs that get into the cytoplasm are translated to produce short peptdies, and in some cases these peptides are stable, and in a few of these cases the peptides have been shown to be functional.  However, function has been shown for only a small fraction of the peptides deriving from lncRNAs. Indeed, the conventional view is that once function has been shown, the transcript is considered an mRNA that was misannotated as a lncRNA. By this convention, no authentic lncRNAs (which are noncoding by definition) code for a functional peptide. Similarly, lines 75-76 might be interpreted to be saying that myriads of lncRNA ORFs are translated into functional peptides, which was not shown in refs 4-7.

The referee is right and we agree with him that it should be the case to re-annotate a ncRNA as coding.

However, many papers are still referring to the unanticipated coding capacities of non coding RNAs (PMID: 31990292 ; PMID: 31836654 ; PMID: 31755573 ; PMID: 28473774), even review PMID: 31907280, showing that the real situation appears to be more complicated.

First, gene names and annotations are not systematically corrected on genomes. It requests some time. Second, some translated non-coding RNAs still carry non-coding functions… and are still named lncRNAs.

The referee is aware that ncRNA were initially predicted as non-coding… because of the lack of long ORF. At present, many of them are described to be associated with ribosomes (and suggested as efficiently translated); it is true that only few peptides were shown to be functional…. Making this field fascinating…

We add new references 4-7 for the first description of pervasive translation of ncRNA.

Line 37. The logic of searching for the conservation of this regulatory feedback loop in plants and animals is flawed. For this logic to make sense, the miRNA pathway itself would have to be conserved from plants to animals. However, the current view is that the miRNA pathways of plants and animals emerged independently, from a common ancestor that had RNAi but not miRNAs.

Thanks for the comment.
Many scientists do not share this point of view since this question is systematically asked in meetings or conferences… I do agree that the current view proposed that the miRNA pathways of plants and animals emerged independently… But it remains a hypothesis.

In contrast, several proteins involved in miRNA processing pathway are conserved…

Line 57. Might be good to say it has been more than 20 years, or to specify the genome that was annotated. Yeast was sequence in 1996.

Thank you for the suggestion. This was corrected p3 lane 57.

Line 91-92. This sentence dismisses the miPEPs reported in humans as not necessarily involved in miRNA expression. However, since miPEP-8 itself does not influence the expression of miR-8, it would seem that the reported activities of the human miPEPs should be described in the introduction and not so readily ignored.

This point was not addressed in these studies and we felt that it was better to discuss these data rather than presenting them as a « state of the art » in the introduction. But we took in account your suggestion and now introduced changes in the introduction from lane 93 p4 to better described their work.

Line 193. The use of "functional ORF" might be misinterpreted to be implying that the peptide is known to be functional. The term "translated ORF" would avoid this confusion.

We introduced this suggestion lane 206

Fig. 5. The RNA-seq analysis seems to be focusing on very small changes with the color-key maxing out at 1.41-fold changes. Also, the analysis of statistical significance is not clear. Which changes are significant (after multiple-hypothesis adjustment)? What is the FDR?

RNA-seq analysis : raw p-values were adjusted with the Benjamini–Hochberg procedure to control the False Discovery Rate (FDR). A gene was declared differentially expressed if it’s adjusted p value ≤ 0.05. Heat map parameters applied: row-by-row normalisation by standardisation (Mean and Standart deviation). These precisions are added to the mat and met line 508 p18 .

Discussion: It would be useful to mention that the molecules that give rise to miR-8 are probably not the same ones that are translated to produce miPEP-8, since Drosha processing would separate the ORF from the poly(A) tail and thereby cause rapid decapping and degradation of the ORF-containing fragment.

We introduced this point in the discussion line 428 p15.

**Second round of review**

**Reviewer 1**

The issue of miPEP-8 expression in S2 cells remains. Indeed, the authors indicate in the reply that "miR-8 is expressed in these cells", and then add the contradictory comment "According to the Western Blot experiments Figure 1 panel D, it seems that the miPEP8 is not expressed at all or only at very low undetectable levels". It is obvious from the blots in Figure 1D that miPEP-8 is not expressed at a detectable level in S2 cells remains. This information should appear in the manuscript.

The author satisfactorily responded to the other issues raised in my previous review

**Reviewer 2**

The authors have satisfactorily addressed most my minor concerns, and they have taken some steps in the direction of addressing my two major concerns, but they are still far from satisfactorily addressing these two concerns.

Major concerns:

1. The interest in this paper rests on the claim that miPEP-8 has a biological function. For this purpose, the results of over-expression studies are a step in the right direction, but not convincing one their own. To convincingly show biological function, the authors need to also perform loss-of-function experiments and observe a loss-of-function phenotype. The authors already seem to have a fly line that lacks the peptide (lines 188-191), but if not, with CRISPR, it is straight-forward to knock out the peptide and look for changes in phenotype and mRNA levels.

>The reviewer is referring to the polymorphisms found in databases or RNA-seq analyses. Unfortunately, we did not have the chance to isolate such a fly stock. We tried several times (during 4 years) to obtain by CRISPR a specific miPEP-8 edited line at the miR-8 locus, even by requesting help from specialized companies. It was not possible to get this edited line. By contrast, as control, it was possible from the first attempt to delete the entire miR-8 locus. We used these lines to develop an alternative strategy by knock in to re-express the pri-miR-8 in miR-8 mutant background using pri-miR-8 wt or pri-miR-8 miPEP8 mutated constructs. miPEP-8 LOF on wings is now provided as well as miR-8 RNA levels Figure 4.

>>It’s a shame the authors were not able to perform a clean loss-of-function experiment. The experiment that they performed (knocking out both miR-8 and miPEP-8 and then adding them back in different combinations) cannot provide a convincing result—especially when the miPEP-8 phenotype is proposed to be a muted version of the miR-8 phenotype. In this experiment, they try to add back the normal level of miR-8, but they have no way of knowing whether they undershoot or overshoot the normal level of miR-8 at the precise developmental time and tissue at which miR-8 specifies wing size. So, although the authors would like to interpret their findings as supporting a function for miPEP-8, the reader is left wondering if this proposed function is simply due to differential expression of miR-8. To investigate the function of miPEP-8 in a more definitive manner, the authors should track down the strain with the polymorphism that removes miPEP-8, backcross this polymorphism into their background, and perform the clean loss-of-function analysis.

2. In the absence of any loss-of-function data, it would be helpful to know if the peptide is conserved any more than expected by chance. Unfortunately, the analysis of peptide conservation provided by the authors is very weak and gives the mistaken impression that the peptide CDS is under negative selection. The analysis of SNPs (lines 178-184) does not give a statistically significant result, and an analysis of conservation among insect or Drosophila whole-genome alignments is missing. Instead, the authors cherry pick the 5 additional Drosophila species in which ATG1 happens to be conserved and show an alignment of the predicted peptides from these species, failing to mention that the peptide is not conserved in any of the other sequenced Drosophila species. Moreover, when one does look at the whole-genome alignments at the UCSC browser, examining the small clade that has the predicted peptide, the pattern of conservation is not what would be expected for a functional peptide. For example, the coding nucleotides are no more conserved than flanking nucleotides, and there is no sign of the triplet periodicity, with more variable silent positions, expected for a peptide CDS under negative selection.

> We are now providing the requested loss-of-function data.

Considering the evolutionary conservation.

- we previously indicated in the text that the miPEP sequence was poorly conserved… We thought that it was most important to present the conserved sequences. We now precise this in the figure legend p22 and in the result section p7 lane 198. We apologize for this oversight.

- We modify the text of the result section (lane 186-188 p7) to present these data as a study and not evidences of miPEP8 existence.

It is true that conservation of ORFs across species can help at identifying functional small proteins that have been preserved by natural selection. However, only few of them have been identified through this way. JP Couso & Patraquim (2017) have proposed in their review that smORF peptides represent very young and rapidly evolving sequences. For example, the sarcolamban (Scl) family of short CDSs show low amino acid sequence conservation, but their structure and molecular function are conserved. One-half of canonical ORFs are conserved across the Kingdom, whereas short CDSs display significant conservation at the Class level. J Ruiz-Orera and M. Mar Albà (2019) also precised in their review that recently evolved de novo proteins are small and poorly conserved across species; therefore, many of them are likely to be missing from the current gene annotations, or being still annotated as noncoding loci.

We believe that the use of experimental methods to identify translated ORFs combined with this analysis represents one important aspect of our study and therefore help to close this gap to improve our understanding of the impact of de novo peptide/proteins in evolutionary innovation.

>>The authors now make statements saying that miPEP-8 is not conserved in some Drosophila species, but they haven’t removed some of their original claims that it is conserved. For example, the section heading is still “Drosophila pri-miR8 encodes a *conserved* miPEP-8, translated in vivo.” Moreover, the SNP analysis, which does not give a statistically significant result, is still in the paper. The authors also still show the alignment of cherry-picked species, now describing the conservation of the peptide in these species as “significant” (line 619), still failing to mention that the pattern of substitutions within these species is not what would be expected for a peptide that is functioning in each of these species. A very young peptide can in principle have an important function, but the authors need to fully come to terms with the fact that the vast majority of young peptides will be disappear in evolutionary time scales before they acquire a function. Therefore, the null hypothesis is that miPEP-8 and other young peptides are non-functional, which is the reason that a clean loss-of-function experiment is so important.

Remaining minor concern:

Line 37. The logic of searching for the conservation of this regulatory feedback loop in plants and animals is flawed. For this logic to make sense, the miRNA pathway itself would have to be conserved from plants to animals. However, the current view is that the miRNA pathways of plants and animals emerged independently, from a common ancestor that had RNAi but not miRNAs.

> Thanks for the comment.

Many scientists do not share this point of view since this question is systematically asked in meetings or conferences… I do agree that the current view proposed that the miRNA pathways of plants and animals emerged independently… But it remains a hypothesis. In contrast, several proteins involved in miRNA processing pathway are conserved…

>> The observation that scientists at conferences ask this question does not mean much. The miRNAs in plants and the miRNAs in animals happen to both be called miRNAs, raising the repeated question by those unfamiliar with the two pathways as to whether they share common ancestry. The answer to this question is that there is little evidence that two pathways diverged from a miRNA pathway of a common ancestor. Instead they appear to have emerged independently from the RNAi pathway. Because the processing proteins shared in the two pathways are also found in the basal RNAi pathway, their presence does not refute the idea that the miRNA pathways of plants and animals emerged independently from a common ancestor that had RNAi, while the processing proteins unique to the two pathways (as well as other differences in biogenesis) support this idea. Even if the authors are enamored with the notion that common ancestry might still be possible, they will avoid losing credibility with the informed reader if they base their logic on the conventional view.

**Authors Response**

**Point-by-point responses to the reviewers’ comments:**

Reviewer #1: The issue of miPEP-8 expression in S2 cells remains.

Indeed, the authors indicate in the reply that "miR-8 is expressed in these cells", and then add the contradictory comment "According to the Western Blot experiments Figure 1 panel D, it seems that the miPEP8 is not expressed at all or only at very low undetectable levels". It is obvious from the blots in Figure 1D that miPEP-8 is not expressed at a detectable level in S2 cells remains. This information should appear in the manuscript.

The following sentence was already introduced lanes 378.

“Such an example was observed in S2 cells in which *miR-8* is expressed at detectable levels whereas endogenous miPEP-8 is not.”

We also add “ Endogenous miPEP-8 expression is undetectable » in the figure legend lane 619.

The author satisfactorily responded to the other issues raised in my previous review

Reviewer #2: The authors have satisfactorily addressed most my minor concerns, and they have taken some steps in the direction of addressing my two major concerns, but they are still far from satisfactorily addressing these two concerns.

Major concerns:
1. The interest in this paper rests on the claim that miPEP-8 has a biological function. For this purpose, the results of over-expression studies are a step in the right direction, but not convincing one their own. To convincingly show biological function, the authors need to also perform loss-of-function experiments and observe a loss-of-function phenotype. The authors already seem to have a fly line that lacks the peptide (lines 188-191), but if not, with CRISPR, it is straight-forward to knock out the peptide and look for changes in phenotype and mRNA levels.

The reviewer is referring to the polymorphisms found in databases or RNA-seq analyses. Unfortunately, we did not have the chance to isolate such a fly stock. We tried several times (during 4 years) to obtain by CRISPR a specific miPEP-8 edited line at the miR-8 locus, even by requesting help from specialized companies. It was not possible to get this edited line. By contrast, as control, it was possible from the first attempt to delete the entire miR-8 locus. We used these lines to develop an alternative strategy by knock in to re-express the pri-miR-8 in miR-8 mutant background using pri-miR-8 wt or pri-miR-8 miPEP8 mutated constructs. miPEP-8 LOF on wings is now provided as well as miR-8 RNA levels Figure 4.

It’s a shame the authors were not able to perform a clean loss-of-function experiment.  The experiment that they performed (knocking out both miR-8 and miPEP-8 and then adding them back in different combinations) cannot provide a convincing result—especially when the miPEP-8 phenotype is proposed to be a muted version of the miR-8 phenotype.  In this experiment, they try to add back the normal level of miR-8, but they have no way of knowing whether they undershoot or overshoot the normal level of miR-8 at the precise developmental time and tissue at which miR-8 specifies wing size. So, although the authors would like to interpret their findings as supporting a function for miPEP-8, the reader is left wondering if this proposed function is simply due to differential expression of miR-8.  To investigate the function of miPEP-8 in a more definitive manner, the authors should track down the strain with the polymorphism that removes miPEP-8, backcross this polymorphism into

their background, and perform the clean loss-of-function analysis.

We followed the reviewer recommandations and analyzed the effect of the miPEP-8 mutation found in DGRP polymorphisms after backcrossing into the white flies background. Homozygotes show reduced wings compared to white control flies. The phenotype is rescued by the re-expression of miPEP-8.

The data have been introduced as a new Fig4 and the text refering to this part lanes 285-299, 455-457, 666-672.

2. In the absence of any loss-of-function data, it would be helpful to know if the peptide is conserved any more than expected by chance. Unfortunately, the analysis of peptide conservation provided by the authors is very weak and gives the mistaken impression that the peptide CDS is under negative selection. The analysis of SNPs (lines 178-184) does not give a statistically significant result, and an analysis of conservation among insect or Drosophila whole-genome alignments is missing. Instead, the authors cherry pick the 5 additional Drosophila species in which ATG1 happens to be conserved and show an alignment of the predicted peptides from these species, failing to mention that the peptide is not conserved in any of the other sequenced Drosophila species. Moreover, when one does look at the whole-genome alignments at the UCSC browser, examining the small clade that has the predicted peptide, the pattern of conservation is not what would be expected for a functional
peptide. For example, the coding nucleotides are no more conserved than flanking nucleotides, and there is no sign of the triplet periodicity, with more variable silent positions, expected for a peptide CDS under negative selection.

We are now providing the requested loss-of-function data.

Considering the evolutionary conservation.
- we previously indicated in the text that the miPEP sequence was poorly conserved… We thought that it was most important to present the conserved sequences. We now precise this in the figure legend p22 and in the result section p7 lane 198. We apologize for this oversight.
- We modify the text of the result section (lane 186-188 p7) to present these data as a study and not evidences of miPEP8 existence.
It is true that conservation of ORFs across species can help at identifying functional small proteins that have been preserved by natural selection. However, only few of them have been identified through this way. JP Couso & Patraquim (2017) have proposed in their review that smORF peptides represent very young and rapidly evolving sequences. For example, the sarcolamban (Scl) family of short CDSs show low amino acid sequence conservation, but their structure and molecular function are conserved. One-half of canonical ORFs are conserved across the Kingdom, whereas short CDSs display significant conservation at the Class level. J Ruiz-Orera and M. Mar Albà (2019) also precised in their review that recently evolved de novo proteins are small and poorly conserved across species; therefore, many of them are likely to be missing from the current gene annotations, or being still annotated as noncoding loci.
We believe that the use of experimental methods to identify translated ORFs combined with this analysis represents one important aspect of our study and therefore help to close this gap to improve our understanding of the impact of de novo peptide/proteins in evolutionary innovation.

The authors now make statements saying that miPEP-8 is not conserved in some Drosophila species, but they haven’t removed some of their original claims that it is conserved. For example, the section heading is still “Drosophila pri-miR8 encodes a *conserved* miPEP-8, translated in vivo.” Moreover, the SNP analysis, which does not give a statistically significant result, is still in the paper. The authors also still show the alignment of cherry-picked species, now describing the conservation of the peptide in these species as “significant” (line 619), still failing to mention that the pattern of substitutions within these species is not what would be expected for a peptide that is functioning in each of these species. A very young peptide can in principle have an important function, but the authors need to fully come to terms with the fact that the vast majority of young peptides will be disappear in evolutionary time scales before they acquire a function. Therefore, the null hypothesis is that miPEP-8 and other young peptides are non-functional, which is the reason that a clean loss-of-function experiment is so important.

We modified the text according the reviewer recommandations : we modified the title of the paragraph, removed the data presenting the SNP analysis and conservation both in text (from lane 185) and figure1.

Remaining minor concern:
Line 37. The logic of searching for the conservation of this regulatory feedback loop in plants and animals is flawed. For this logic to make sense, the miRNA pathway itself would have to be conserved from plants to animals. However, the current view is that the miRNA pathways of plants and animals emerged independently, from a common ancestor that had RNAi but not miRNAs.

Thanks for the comment.

Many scientists do not share this point of view since this question is systematically asked in meetings or conferences… I do agree that the current view proposed that the miRNA pathways of plants and animals emerged independently… But it remains a hypothesis. In contrast, several proteins involved in miRNA processing pathway are conserved…

The observation that scientists at conferences ask this question does not mean much. The miRNAs in plants and the miRNAs in animals happen to both be called miRNAs, raising the repeated question by those unfamiliar with the two pathways as to whether they share common ancestry. The answer to this question is that there is little evidence that two pathways diverged from a miRNA pathway of a common ancestor. Instead they appear to have emerged independently from the RNAi pathway. Because the processing proteins shared in the two pathways are also found in the basal RNAi pathway, their presence does not refute the idea that the miRNA pathways of plants and animals emerged independently from a common ancestor that had RNAi, while the processing proteins unique to the two pathways (as well as other differences in biogenesis) support this idea. Even if the authors are enamored with the notion that common ancestry might still be possible, they will avoid losing credibility with

the informed reader if they base their logic on the conventional view.

**Third round of review**

Reviewer 2:

The authors have satisfactorily addressed my major concerns. After reading through their revised text, I have just a few minor corrections:

1. Line 37. Change “conserved” to “present.” This correction would address my original minor concern regarding the problems with implying that plant and animal miRNAs did not arise independently.

2. Lines 51 and 88. Change “microRNA encoded” to “pri-microRNA-encoded.” MicroRNAs do not encode peptides. It’s the pri-microRNAs that encode peptides.

3. Line 61. Insert a comma after “(miR)” to avoid implying that microRNAs silence transposons.

4. Lines 85-88. We have long known that a large fraction of metazoan pri-miRNAs code for proteins. For example, there are many cases in which miRNAs are processed from the introns of pre-mRNAs, and there are also cases in which miRNAs are processed from mature mRNAs. In all of these cases, the pri-miRNAs are also pre-mRNAs. Therefore, the discovery of miPEPs does not overturn a dogma, it merely adds a few more instances in which pri-miRNAs are also pre-mRNAs. These sentences should be revised accordingly.

5. Lines 214-217 and 299-300. These lines are missing figure citations.

6. Lines 350-351. Some transcripts that are misannotated as lncRNAs (including pri-miRNAs that produce miPEPs) can encode peptides. Because they encode peptides, these misannotated lncRNAs are actually mRNAs. Many other classes of RNAs (e.g., tRNAs, snRNA, snoRNAs, rRNAs, RNaseP RNA, telomerase RNA, …) cannot produce peptides. So, it is not correct to say that “virtually all classes of RNAs can produce these peptides.”

7. Line 405. The use of the word “conserved” implies that that the drosophila miPEP-8 and mammalian miPEP-200a/b are conserved, which cannot be true, given the recent origin of drosophila miPEP-8.

8. Lines 418-420. Because these pri-miRs code for peptides, by definition, they come from protein-coding genes, and thus it would be more correct to end the sentence with “… for other coding genes.”

**Authors Response**

**Point-by-point responses to the reviewers’ comments:**

Reviewer #2: The authors have satisfactorily addressed my major concerns. After reading through their revised text, I have just a few minor corrections:

1. Line 37.  Change “conserved” to “present.” This correction would address my original minor concern regarding the problems with implying that plant and animal miRNAs did not arise independently.

Line 37 « conserved » has been changed to « present »

2. Lines 51 and 88. Change “microRNA encoded” to “pri-microRNA-encoded.”  MicroRNAs do not encode peptides. It’s the pri-microRNAs that encode peptides.

Corrected
3. Line 61. Insert a comma after “(miR)” to avoid implying that microRNAs silence transposons.

Corrected

4. Lines 85-88. We have long known that a large fraction of metazoan pri-miRNAs code for proteins.  For example, there are many cases in which miRNAs are processed from the introns of pre-mRNAs, and there are also cases in which miRNAs are processed from mature mRNAs. In all of these cases, the pri-miRNAs are also pre-mRNAs. Therefore, the discovery of miPEPs does not overturn a dogma, it merely adds a few more instances in which pri-miRNAs are also pre-mRNAs.  These sentences should be revised accordingly.

We changed the sentence to :

However, the discovery that plant *pri-miR*s encode small regulatory peptides merely adds evidences that *pri-miRNAs* are also pre-mRNAs

5. Lines 214-217 and 299-300. These lines are missing figure citations.

Corrected

6. Lines 350-351. Some transcripts that are misannotated as lncRNAs (including pri-miRNAs that produce miPEPs) can encode peptides. Because they encode peptides, these misannotated lncRNAs are actually mRNAs. Many other classes of RNAs (e.g., tRNAs, snRNA, snoRNAs, rRNAs, RNaseP RNA, telomerase RNA, …) cannot produce peptides. So, it is not correct to say that “virtually all classes of RNAs can produce these peptides.”

We changed to « many classes of RNAs can produce these peptides”

7. Line 405.  The use of the word “conserved” implies that that the drosophila miPEP-8 and mammalian miPEP-200a/b are conserved, which cannot be true, given the recent origin of drosophila miPEP-8.
« conserved » has been changed to « present »

8. Lines 418-420. Because these pri-miRs code for peptides, by definition, they come from protein-coding genes, and thus it would be more correct to end the sentence with “… for other coding genes.”

« other » inserted
